# Causal Wavelet Residual Learning with Observation-Support Gating for Industrial Sensor Anomaly Detection

**DOI:** 10.3390/s26185751

**Published:** 2026-09-10

**Authors:** Shunyao Teng, Xiang Gao

**Affiliations:** 1 Haide College, Ocean University of China, Qingdao 266100, China; tengshunyao@stu.ouc.edu.cn; 2 School of Mathematical Sciences, Ocean University of China, Qingdao 266100, China

**Keywords:** industrial sensors, anomaly detection, multivariate time series, wavelet residuals, false alarms, causal prediction, leakage-safe evaluation

## Abstract

Industrial sensor anomalies must be detected under missingness, noise, drift, and finite operator attention. Point-wise scores alone can obscure the resulting alarm burden. We present RC-WMRAD, a causal wavelet residual detector that combines raw temporal prediction with a left-padded multi-scale Haar path and an observation-support gate. Training uses normal data, thresholds are calibrated independently, score bundles are locked before labels are read, and evaluation uses no point adjustment. Six public benchmarks reveal conditional effects. Against Raw-TCN, RC-WMRAD reduces false alarms on SKAB, SMD, MSL, SMAP, and the railway MetroPT-3 benchmark, but the accompanying ranking and event-coverage effects differ by dataset. Against TranAD-style, SMD shows lower AUPRC and higher event recall, whereas PSM shows lower event recall and uncertain AUPRC. On MetroPT-3, TranAD-style ranks failures more strongly, covers a larger fraction of reported failure intervals, and responds earlier, while RC-WMRAD produces fewer false-alarm segments. Corruption, ablation, and calibration analyses preserve these metric-specific boundaries rather than establishing universal robustness. The study therefore contributes an auditable sensor-monitoring protocol and a scoped account of ranking, partial event coverage, and alarm burden, with no pooled global effect.

## 1. Introduction

Industrial monitoring systems collect multivariate streams from pumps, valves, servers, water loops, spacecraft, and other engineered processes. An anomaly detector in these settings must handle faults alongside missing observations, noise, drift, and operating-regime changes. Frequent alarms under ordinary corruption can prevent deployment even when point-wise recall is high. Evaluation should therefore include false alarms per hour, detection delay, event recall, and the provenance of the threshold that converts a continuous score into an alarm.

An industrial alarm is not merely a binary classification output. It is an intervention request delivered to an operator, maintenance system, or supervisory controller. The cost of a detector therefore depends on at least three coupled properties: whether abnormal samples receive high scores, whether temporally extended fault events are covered, and how often normal operation is fragmented into alert segments. A method can improve one property while degrading another. This distinction is especially important when anomalous samples are rare, because AUROC can remain favorable while precision and operator-facing burden deteriorate [1,2,3]. Our evaluation consequently separates ranking metrics from a locked operating point and from event-level alarm behavior.

Two practical constraints motivate this work. First, sensor benchmarks often provide long chronological recordings rather than independent samples. Whole-file normalization, transformation, or threshold selection can introduce future information or label knowledge. Second, high recall can be operationally unacceptable when alarm segments are too dense for operators or supervisory systems. These constraints require both causal feature construction and a separation between score generation and label-based evaluation.

The first constraint concerns information flow rather than model class. A nominally online architecture can still leak future information if centered filtering, symmetric wavelet extension, backward filling, whole-file normalization, or label-conditioned threshold selection is performed outside the network. The second concerns decision semantics. Point adjustment, in which detection of one point may credit an entire anomaly interval, can conceal late detection and alarm fragmentation [4,5,6]. We therefore regard causal preprocessing, score locking, independent calibration, and non-point-adjusted evaluation as components of the method contract rather than post-processing details.

Wavelet representations can expose transient and persistent deviations at different temporal scales [7,8,9], but an offline transform may use samples that have not yet arrived. Thresholds selected from test labels can further inflate reconstruction-based results. Prior work has explored wavelet graph autoencoders, graph-attention models, stochastic recurrent models, one-class objectives, and Transformer discrepancies [10,11,12,13,14,15]. Recent hyperspectral methods such as HELWC and S3CRAD also illustrate decomposition, local contrast, and explicit background modeling, although their spatial–spectral setting is not a direct causal time-series comparator [16,17]. Haar filters, causal convolutions, positive residual scales, masks, and learned fusion are individually established ideas; the claimed contribution is not a new anomaly-detection primitive. The relevant question is whether their timestamp-aligned, leakage-audited integration changes deployment-facing trade-offs under a fixed evaluation contract.

We present RC-WMRAD, a causal wavelet multi-scale residual detector with observation-support gating. A raw temporal predictor and a left-padded causal Haar path produce one-step residual evidence after the target observation arrives. Chronological training, validation, calibration, and test roles are fixed. Preprocessing uses normal training data, thresholds use normal-calibration scores, score bundles are locked before labels are read, and primary metrics use no point adjustment. The identifier RC-WMRAD is retained for continuity with the implementation and audit artifacts; it is not used as a claim of probabilistically calibrated reliability.

Two mechanisms must be distinguished. First, a learned observation-support gate uses history availability to interpolate raw and wavelet residual evidence; it estimates neither epistemic uncertainty nor probability of correct detection. Second, an external normal-only calibration set maps the scalar score to a fixed empirical threshold. This threshold is an operating rule rather than a distribution-free reliability guarantee. Probabilistic scale calibration, decision-threshold calibration, and robustness to distribution shift remain different properties [18,19,20].

The evidence is interpreted at its admitted statistical unit. It includes five-seed SKAB and PSM comparisons, 28-entity SMD, 27-channel MSL, and 53-channel SMAP evaluations. The study establishes no universal superiority and no universal robustness. Instead, it tests when ranking quality, event coverage, and alarm burden move together or in opposite directions. This framing turns apparently contradictory results into reportable evidence. For example, a reduction in false alarms can be operationally desirable while a simultaneous loss of event recall is unacceptable for safety-critical monitoring. Conversely, a detector can recover more events at the expense of a denser alarm stream. Rather than compressing these outcomes into a single cross-dataset average, we preserve comparator, dataset, seed, entity, corruption, and metric identities.

The study makes the following contributions:

1. An implementation-audited integration of causal raw and wavelet residual paths with explicit one-step target handling, left-aligned Haar coefficients, scale-normalized residuals, and fail-closed masking;
2. A leakage-safe protocol separating model selection, normal calibration, opaque score generation, score locking, and label evaluation;
3. A cross-dataset study of metric-specific effects against matched Raw-TCN and protocol-matched TranAD-style comparators;
4. Robustness, calibration, event-attribution, alarm-burden, and complexity audits that retain negative and inconclusive evidence.

These contributions are empirical and protocol-specific. They do not assert that Haar coefficients dominate learned temporal features, that the observation-support gate identifies physical sensor reliability, or that the selected calibration quantile is universally optimal. Their value lies in the leakage-safe integration and auditable separation of representation, scoring, calibration, and deployment-facing evaluation.

## 2. Related Work

### 2.1. Multivariate Time-Series Anomaly Detection

Anomaly detection methods identify observations that depart from an estimated notion of normality [21,22]. Classical approaches include local-density estimators and random partitioning methods such as LOF and Isolation Forest [23,24]. Deep one-class methods instead learn representations that concentrate normal samples in a compact region [25], while probabilistic and reconstruction models use likelihood, energy, or reconstruction discrepancy [26]. These families provide useful conceptual baselines, but they do not by themselves specify how chronological sensor data, missing observations, and event-level alerts should be handled.

Multivariate time-series detectors add temporal and cross-variable structure. MSCRED reconstructs multi-scale inter-sensor signature matrices [27]; OmniAnomaly uses a stochastic recurrent model with reconstruction probabilities [12]; USAD combines autoencoding and adversarial training [28]; and InterFusion uses a hierarchical variational model to separate inter- and intra-variable dependencies [29]. Graph approaches explicitly model sensor dependencies through attention or learned graphs [30,31]. Temporal one-class and contrastive methods provide further alternatives to direct reconstruction [13,32].

Transformer detectors model long-range temporal structure or association discrepancy. TranAD uses self-conditioning and adversarial training, whereas Anomaly Transformer contrasts learned associations with a prior association structure [14,15]. These models are important comparators because they can produce strong point-wise anomaly rankings, but a ranking architecture does not determine a deployment threshold. Moreover, architecture labels alone do not guarantee causal preprocessing or matched label access. Our comparator protocol therefore fixes input history, mask access, normal-only calibration, score locking, and the evaluator around each model.

Prediction-based methods estimate the next observation from a past window, whereas reconstruction methods encode and recover an observed window. Prediction naturally exposes a temporal boundary: a target cannot contribute to its own predictor. Reconstruction can be equally valid, but its masking, receptive field, and score alignment must be audited carefully. RC-WMRAD follows the normal-only predictive-residual tradition and uses causal temporal convolutions, whose long effective receptive fields can be obtained without recurrence [33]. It keeps raw and wavelet evidence separate until score fusion so that matched post-primary paths can identify their event-level contributions.

### 2.2. Wavelet and Time–Frequency Detectors

Wavelet analysis represents a signal using localized low- and high-frequency responses across scales. Multiresolution analysis and perfect-reconstruction filter-bank theory provide the mathematical foundation for separating coarse behavior from localized changes [7,8,9]. In anomaly detection, this separation is attractive because abrupt faults, oscillations, slow drifts, and regime transitions need not occupy the same temporal scale.

Recent detectors combine wavelet features with learned cross-variable models. MEGA uses multi-scale wavelet processing with graph autoencoding [10], while discrete-wavelet decomposition and dual graph attention have been studied for multivariate IoT monitoring [11]. These approaches establish that wavelet processing itself is not a sufficient novelty claim. RC-WMRAD instead focuses on three narrower properties: every coefficient is left-aligned to its score timestamp, predictive residuals retain explicit scale normalization, and the alarm stream is evaluated after an independently locked threshold.

Offline wavelet transforms may use symmetric extension, centered filters, or full-file context. Such operations are appropriate for retrospective analysis but not for streaming scores attached to time *t*. Even an undecimated transform, which preserves temporal resolution, is not automatically causal: boundary handling and coefficient alignment still determine whether future samples enter a coefficient. The implementation therefore uses a left-padded, undecimated Haar filter bank with dilations $1,2,\ldots ,2^{J-1}$. It is not a general stationary wavelet transform, wavelet packet decomposition, learned filter bank, or adaptive wavelet method.

Industrial and cross-domain sensing studies provide complementary context but not protocol-matched time-series baselines. Petroleum sensor analysis illustrates the importance of application-specific feature validation and anomaly interpretation [34]. In hyperspectral anomaly detection, HELWC combines empirical-mode decomposition with local contrast, HLSC-SSGF emphasizes local spatial–spectral gradients, DFBSNet separates and fuses frequency branches, and S3CRAD combines background reconstruction with spatial–spectral constraints [16,17,35,36]. DSCH-Net likewise uses physics-inspired, direction-aware representation under heterogeneous imaging degradation [37]. These image-domain methods concern spatial or spectral backgrounds rather than causal multivariate sensor streams; we cite them only as complementary evidence that decomposition, discriminative local contrast, and explicit background assumptions must be validated under the operating domain.

### 2.3. Observation Support and Calibration

Reliability can refer to data availability, predictive uncertainty, score stability, or calibrated decision risk; these quantities should not be conflated. Heteroscedastic prediction models separate a location estimate from an input-dependent scale, but a predicted scale is not automatically calibrated under shift [19,38]. Classification calibration likewise shows that accurate neural networks can produce unreliable confidence values [18]. Dynamic anomaly thresholds can incorporate uncertainty explicitly, but introduce additional assumptions and tuning choices [20].

RC-WMRAD uses a more modest signal: the fraction of observed history and the availability of the most recent sample. The gate therefore describes observation support, not epistemic uncertainty or fault probability. A related design principle appears in object-aware active 3D reconstruction, where uncertainty is restricted to task-relevant structure [39]. OUGS concerns view selection for 3D Gaussian Splatting rather than time-series detection, so we cite it only as an example of targeted, interpretable uncertainty use. Robustness remains an empirical outcome here because gains under one corruption can coexist with failure under another.

### 2.4. Evaluation Protocols and Alarm Metrics

Time-series benchmarks are sensitive to label quality, anomaly position, thresholding, and point adjustment [5,6,40]. Range-aware evaluation formalizes that anomaly events have duration and that early, complete, and non-fragmented detection may have different value [4]. Local evaluation further shows that aggregate scores can hide where a detector succeeds or fails [6]. These observations motivate retaining point ranking, event coverage, false-alarm segments, and first-hit delay as separate outputs.

Point adjustment can turn a late or fragmented alert into apparent event recovery and can make a permissive threshold appear effective. We instead report non-point-adjusted rank, event, alarm, and delay metrics. AUPRC is emphasized because anomalous timestamps are rare and precision is sensitive to false positives, while AUROC remains a threshold-free discrimination summary [1,2,3]. Calibration thresholds use normal scores only, and labels are read only after score bundles are locked. Bootstrap intervals preserve the paired seed and entity structure rather than treating every timestamp as an independent replicate [41,42].

## 3. Materials and Methods

### 3.1. Problem Setup

Consider one monitored entity with *D* sensor variables sampled at ordered times t=1,…,T. Its value, observation mask, and evaluation label are

(1) $$x_{t}={\left(x_{t,1},\ldots ,x_{t,D}\right)}^{⊤}\in R^{D},\;m_{t}\in {\left\{0,1\right\}}^{D},\;y_{t}\in \left\{0,1\right\},$$

where $m_{t,v}=1$ means that variable *v* is observed. Labels are not model inputs and are unavailable during training, calibration, and test-score generation. They are introduced only by the final evaluator.

For history length L=128, define the one-step input ending at time *t* as

(2) $$H_{t}=\left\{\left(x_{t-L+1},m_{t-L+1}\right),\ldots ,\left(x_{t},m_{t}\right)\right\}.$$

The model maps $H_{t}$ to predictive parameters for target time t+1. The filtration $F_{t}$ contains training-derived constants and all observations available no later than *t*. A valid pre-target prediction must satisfy

(3) $$\left(\mu_{t+1},\sigma_{t+1}\right)=f_{\theta }\left(H_{t}\right),\;f_{\theta }\left(H_{t}\right)\;\mathrm{is}\;F_{t}\text{-}\mathrm{measurable}.$$

The target $x_{t+1}$ forms residual evidence only after arrival. If $\sum_{v}m_{t+1,v}=0$, the timestamp is unscorable rather than silently classified as normal.

This setup separates prediction from scoring. A large forecast error may indicate a process fault, an unseen regime, sensor corruption, or an inadequately estimated residual scale. The detector does not identify physical fault type; it produces evidence for a downstream alarm rule.

### 3.2. Train-Only Preprocessing

Let $I_{v}^{\mathrm{tr}}$ be the indices at which variable *v* is observed in the normal training partition. Coordinate-wise location is fitted as the training median,

(4) $${\hat{a}}_{v}=\mathrm{median}\left\{x_{t,v}:t\in I_{v}^{\mathrm{tr}}\right\}.$$

The implementation uses a Gaussian-consistent median absolute deviation (MAD), with mean absolute deviation as a zero-MAD fallback and a strictly positive numerical floor:

(5) $${\hat{b}}_{v}=\max\;\left[1.4826\left\{\begin{array}{cc} {\mathrm{median}}_{t\in I_{v}^{\mathrm{tr}}}\left|x_{t,v}-{\hat{a}}_{v}\right|, & {\mathrm{MAD}}_{v}>0,\\ {\mathrm{mean}}_{t\in I_{v}^{\mathrm{tr}}}\left|x_{t,v}-{\hat{a}}_{v}\right|, & {\mathrm{MAD}}_{v}=0, \end{array}\right.10^{-6}\right].$$

Robust location and scale reduce sensitivity to isolated training extremes, but they do not make the procedure immune to contaminated training data [43]. Every variable must contain at least one observed normal-training value; otherwise preprocessing fails closed.

Observed values are standardized using only $\left({\hat{a}}_{v},{\hat{b}}_{v}\right)$. The preprocessing routine first forms a causal forward-filled state from the most recent standardized observation:

(6) $$\begin{array}{cc} {\tilde{z}}_{t,v}=\frac{x_{t,v}-{\hat{a}}_{v}}{{\hat{b}}_{v}},\;\; & m_{t,v}=1, \end{array}$$

(7) $$\begin{array}{cc} z_{t,v}^{\mathrm{ff}}=m_{t,v}{\tilde{z}}_{t,v}+\left(1-m_{t,v}\right)z_{t-1,v}^{\mathrm{ff}},\;\; & z_{0,v}^{\mathrm{ff}}=0. \end{array}$$

At the model boundary, both predictive branches receive the same mask-controlled value tensor

(8) $$u_{t,v}=m_{t,v}z_{t,v}^{\mathrm{ff}},$$

so an unobserved position is explicitly represented by zero while the original binary mask remains available to the raw branch and the observation-support gate. Consequently, forward filling is a causal preprocessing state, but a filled value is not consumed as if it were observed. This two-stage description resolves the apparent difference between forward filling and masked zero substitution: the former avoids future interpolation, and the latter is the actual branch input at missing positions. No backward filling, interpolation from future samples, test-statistic normalization, or label-informed filtering is used. Statistics are fitted once on normal training data and then frozen for validation, calibration, and test partitions.

### 3.3. Causal Wavelet Residual Path

The Haar low- and high-pass kernels are

(9) $$h=2^{-1/2}\left(1,1\right),\;g=2^{-1/2}\left(-1,1\right).$$

For level j∈{1,…,J}, dilation is $d_{j}=2^{j-1}$. Applied to the masked standardized tensor with left padding, the aligned approximation and detail coefficients are

(10) $$\begin{array}{c} c_{t,v,j}^{\mathrm{lo}}=2^{-1/2}\left(u_{t-d_{j},v}+u_{t,v}\right), \end{array}$$

(11) $$\begin{array}{c} c_{t,v,j}^{\mathrm{hi}}=2^{-1/2}\left(-u_{t-d_{j},v}+u_{t,v}\right), \end{array}$$

where out-of-window left indices receive zero padding. No decimation is performed, so every level remains aligned to every target timestamp. The multi-scale vector for variable *v* is

(12) $$c_{t,v}=\left(c_{t,v,1}^{\mathrm{lo}},c_{t,v,1}^{\mathrm{hi}},\ldots ,c_{t,v,J}^{\mathrm{lo}},c_{t,v,J}^{\mathrm{hi}}\right)\in R^{2J}.$$

Causality follows directly from the support of Equations (10) and (11): each coefficient at *t* depends on $\left\{u_{t-d_{j},v},u_{t,v}\right\}$ and hence on indices no greater than *t*. Stacking levels and applying causal convolutions preserves this dependency,

(13) $$\mathrm{supp}\left(c_{t,v}\right)\subseteq \left\{t-2^{J-1},\ldots ,t\right\}\subseteq F_{t}.$$

This is an information-dependency statement, not a claim that the representation is optimal.

The admitted checkpoints use J=3 for SKAB and PSM and J=2 for SMD, MSL, SMAP, and MetroPT-3. These bounded settings were fixed before the reported test-score bundles: the three-level setting retains an additional scale in the earlier SKAB/PSM development path, whereas the two-level setting limits the variable–band output dimension in the multi-entity and external protocols. They were not reselected by corruption severity, reported metric, or test label. An exploratory five-seed PSM sweep over J∈{1,2,3,4} did not identify a level that jointly dominated ranking and event recall; it is therefore a sensitivity analysis, not evidence that either admitted value is universally optimal.

The raw branch receives the concatenation of u and its mask. The wavelet branch receives multi-level coefficients computed from the same u tensor. Separate 1×1 projections and left-padded causal convolutional blocks produce final hidden states $h_{t}^{x}$ and $h_{t}^{w}$. In the admitted independent-branch model,

(14) $$\left(\mu_{t+1}^{x},\rho_{t+1}^{x}\right)=q_{x}(h_{t}^{x}),\;\left(\mu_{t+1}^{w},\rho_{t+1}^{w}\right)=q_{w}(h_{t}^{w}),$$

where the wavelet outputs are indexed by variable and by 2J bands. Positive residual scales are

(15) $$\sigma_{t+1}^{x}=\mathrm{softplus}\left(\rho_{t+1}^{x}\right)+10^{-3},\;\sigma_{t+1}^{w}=\mathrm{softplus}\left(\rho_{t+1}^{w}\right)+10^{-3}.$$

The scale floor prevents division by zero but does not guarantee probabilistic calibration.

### 3.4. Observation-Support-Gated Score and Objective

After $x_{t+1}$ arrives, it is standardized and appended to the causal history solely to compute the aligned target coefficient $c_{t+1,v}$. The raw and mean band residuals are

(16) $$\begin{array}{c} r_{t+1,v}^{x}=\left|\frac{z_{t+1,v}-\mu_{t+1,v}^{x}}{\sigma_{t+1,v}^{x}}\right|, \end{array}$$

(17) $$\begin{array}{c} r_{t+1,v}^{w}=\frac{1}{2J}\sum_{k=1}^{2J}\left|\frac{c_{t+1,v,k}-\mu_{t+1,v,k}^{w}}{\sigma_{t+1,v,k}^{w}}\right|. \end{array}$$

Both residuals are dimensionless. The raw residual asks whether the next value is surprising relative to the raw predictor; the wavelet residual asks whether its aligned multi-scale response is surprising. Averaging bands prevents the wavelet score magnitude from growing mechanically with *J*.

For each variable, history availability and most recent availability are

(18) $${\bar{m}}_{t,v}=\frac{1}{L}\sum_{\ell =0}^{L-1}m_{t-\ell ,v},\;\gamma_{t,v}=\mathrm{sigmoid}\left(w_{g}^{⊤}\left[{\bar{m}}_{t,v},m_{t,v}\right]+b_{g}\right).$$

Thus, $0<\gamma_{t,v}<1$. This gate is a learned interpolation conditioned on observation support. It is not a complete noise, drift, or uncertainty estimator, and a high value should not be interpreted as a probability that the raw branch is correct.

The observed-target variable score is

(19) $$s_{t+1,v}=m_{t+1,v}\left[\gamma_{t,v}r_{t+1,v}^{x}+\left(1-\gamma_{t,v}\right)r_{t+1,v}^{w}\right].$$

Multiplication by $m_{t+1,v}$ is fail-closed: a non-finite missing target is replaced before arithmetic and then contributes no score. Every formal benchmark result in this manuscript uses the arithmetic mean over the *D* variable scores,

(20) $$S_{t+1}=\frac{1}{D}\sum_{v=1}^{D}s_{t+1,v},$$

after zeroing unobserved targets as in Equation (19); a timestamp with no observed target variables is rejected as unscorable. Thus, the aggregation is not selected separately for SKAB, SMD, PSM, MSL, SMAP, or MetroPT-3, and the same rule is used for both members of every formal comparison. It is fixed before labels are opened.

The v1 executable training path underlying every benchmark result reported in this manuscript minimizes the same gate-weighted standardized absolute residual used before timestamp aggregation. For the observed target pairs 𝒪 in a batch,

(21) $$L_{v1}\left(\theta \right)=\frac{1}{\left|𝒪\right|}\sum_{(t+1,v)\in 𝒪}\left[\gamma_{t,v}r_{t+1,v}^{x}+\left(1-\gamma_{t,v}\right)r_{t+1,v}^{w}\right].$$

The branches therefore are not assigned fixed one-half loss weights: their contribution is observation-support gated for each variable and window. Band averaging in Equation (17) prevents the wavelet term from scaling mechanically with *J*, but the gate-weighted objective is a transparent implementation choice rather than a proven optimum. If a batch contains no observed target, it is handled fail-closed rather than assigned an artificial zero loss. The same v1 objective is used for validation and model-health checks; thresholds and test labels do not enter optimization. Because Equation (21) contains no likelihood log-scale penalty, the positive scales in Equation (15) are operational score normalizers rather than identifiable or calibrated predictive standard deviations. A versioned gate-weighted Gaussian negative-log-likelihood path with an explicit log-scale term has been implemented as a method-integrity candidate, but it is not used to produce any numerical result in this manuscript. Promotion of that path requires a stable, audited multi-benchmark retraining chain; until then, the reported evidence remains explicitly tied to $L_{v1}$.

**Figure 1:**
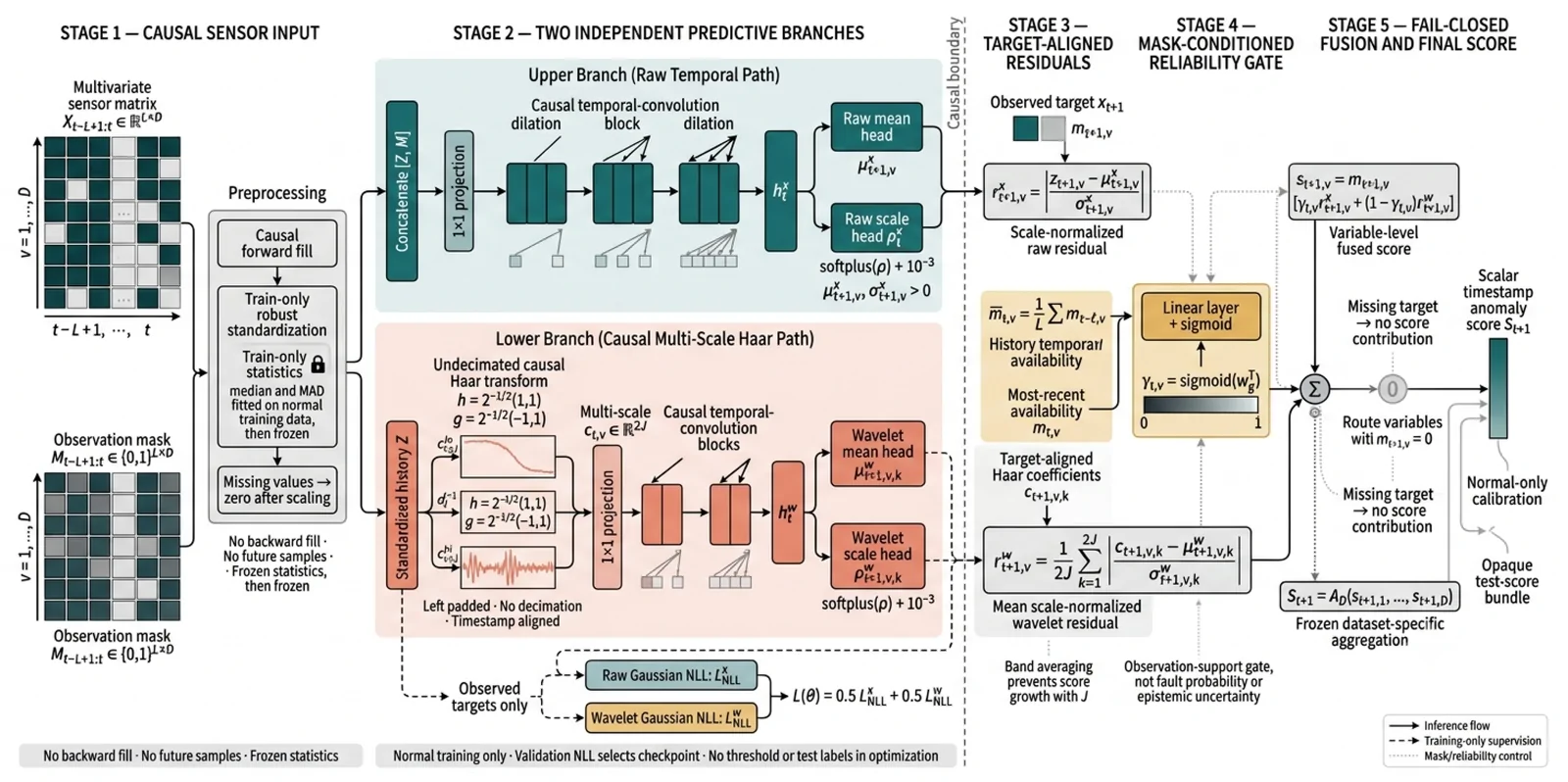
**RC-WMRAD architecture and score construction.** A causally preprocessed value–mask history enters independent raw temporal and undecimated Haar branches. Scale-normalized residuals are fused by an observation-support gate, target-masked, and aggregated into a timestamp score. Dashed paths denote normal-only supervision; the target does not feed either predictor.

Figure 1 summarizes the complete causal computation. It separates information available at prediction time from the arrived target used only to form residuals and training supervision, while exposing the mask-controlled route from branch scores to the final timestamp score.

### 3.5. Score Locking and Label Access

Let $C={\left\{S_{i}^{\mathrm{cal}}\right\}}_{i=1}^{n_{c}}$ be finite scores from the independent normal-calibration partition. With q=0.995, the threshold is the higher empirical quantile

(22) $$\tau_{q}=S_{\left(\left\lceil qn_{c}\right\rceil \right)}^{\mathrm{cal}},\;q=0.995,$$

where the parenthesized subscript denotes the ordered calibration score. A timestamp is alerting only when

(23) $${\hat{y}}_{t}=I\left(S_{t}>\tau_{q}\right);$$

equality is non-alerting. The threshold is therefore reproducible from normal-calibration scores and a declared quantile rule. It is not optimized against test F1.

The pipeline order is

(24) $$\begin{array}{cc} \mathrm{normal}\;\mathrm{fit} & \to \mathrm{checkpoint}\;\mathrm{selection}\to \mathrm{normal}\;\mathrm{calibration}\;\mathrm{scores}\to \tau_{q},\\ \tau_{q} & \to \mathrm{opaque}\;\mathrm{test}\;\mathrm{scores}\to \mathrm{hash}\;\mathrm{lock}\to \mathrm{label}\;\mathrm{evaluation}. \end{array}$$

Test bundles contain entity/timestamp keys and scores but no labels, alerts, or metrics. Calibration and test arrays receive SHA-256 digests before labels are opened. Admissible results link training, preprocessing, calibration, scores, thresholds, label alignment, and statistical units through non-overwriting manifests. This ordering cannot eliminate all research degrees of freedom, but it prevents a common direct path from test labels to the reported operating threshold.

**Figure 2:**
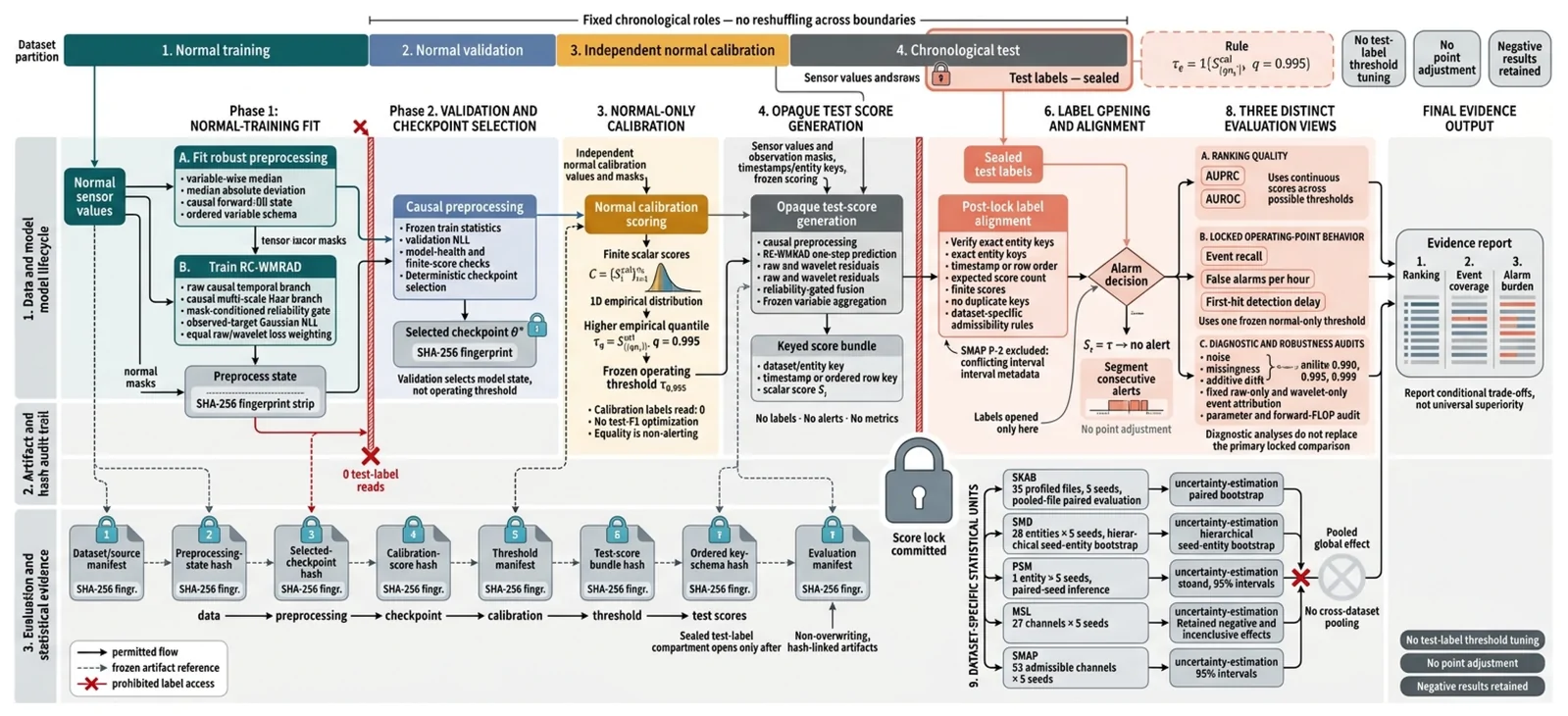
**Leakage-safe evidence workflow.** Chronological fitting and validation precede independent normal calibration; opaque keyed test scores are hash-locked before labels are opened. Post-lock evaluation separates ranking quality, event coverage, and alarm burden, while retaining dataset-specific statistical units without point adjustment or cross-dataset pooling.

Figure 2 makes the separation between model development, calibration, opaque score generation, and post-lock evaluation explicit. The lock is procedural rather than cryptographic access control: its role is to leave an auditable record that threshold choice and score generation preceded label-dependent analysis.

## 4. Experimental Protocol

### 4.1. Datasets and Evaluation Units

The study uses SKAB v0.9 [44], SMD [12], PSM [45], the MSL/SMAP spacecraft telemetry benchmarks introduced for operational anomaly detection [46], and MetroPT-3 [47,48]. MetroPT-3 contributes 1,516,948 chronological measurements from 15 pressure, temperature, current, and digital-control signals in a railway air-production unit. Its first calendar month is divided chronologically into normal training, validation, and calibration; later records form the sealed test stream. The four company failure-report intervals define events. A fixed one-minute decision cadence is used while every decision retains the preceding 128 original samples, whose median spacing is 10 s. These datasets differ materially in acquisition process, dimensionality, event duration, and unit of replication. SKAB comprises 35 profiled files and is evaluated as a pooled-file, paired-seed benchmark. SMD contributes 28 entities, PSM one entity, MSL 27 channels, and SMAP 53 admissible channels. SMAP channel P-2 is excluded because its metadata contain conflicting anomaly intervals. Table 1 keeps these evaluation units separate.

All partitions are chronological. Training establishes model parameters and robust preprocessing statistics; validation selects checkpoints; normal calibration fixes the operating threshold; and testing measures locked scores. No dataset is used to estimate a common cross-dataset normalization or common threshold. This separation is important because a numerical score has no intrinsic meaning across architectures or entities until its calibration provenance is specified.

### 4.2. Comparators and Model Components

Raw-TCN is the matched architectural control. It uses the same history, one-step horizon, preprocessing, independent calibration, and evaluator. Its causal temporal-convolution backbone follows the general sequence-modeling design studied by Bai et al. [33], but its width and output heads are fixed by this repository’s comparison contract. The control isolates the effect of adding the causal Haar branch and learned fusion while keeping the deployment pipeline unchanged.

TranAD-style is a clean-room causal Transformer comparator with the same value-and-mask history and score-lock protocol [14]. It is parameter-comparable to RC-WMRAD but not forward-compute matched. The designation TranAD-style is deliberate: the implementation preserves the relevant Transformer comparison boundary without claiming byte-level identity to the authors’ original software environment. Two complementary component analyses are kept separate. Fixed raw-only and wavelet-only SMD scores are post-primary rescoring paths from frozen RC-WMRAD checkpoints; they localize which frozen residual source covers an event but are not independent model-training comparisons. The SKAB ablation instead independently optimizes A0–A5 for each of five seeds under identical chronological fitting, checkpoint selection, normal-only calibration, and label-gated evaluation.

MetroPT-3 additionally includes two clean-room baseline families. USAD-style retains the dual-decoder adversarial reconstruction structure of USAD [28]; GDN-style learns node embeddings, a top-*k* sensor graph, and graph-conditioned next-step predictions following the central GDN motivation [31]. The “-style” suffix is essential: these repository implementations preserve the named modeling boundary and common data-access protocol but do not claim byte-identical reproduction of upstream software or dataset-specific hyperparameter searches. Table 2 summarizes which raw, wavelet, mask, and fusion components each comparator or score path audits.

### 4.3. Thresholds, Events, and Statistical Scope

The primary operating point is the 99.5th percentile of normal-calibration scores from Equation (22). Equality is non-alerting. Let $E={\left\{\left[a_{k},b_{k}\right]\right\}}_{k=1}^{K}$ denote maximal contiguous truth events and $\hat{E}={\left\{\left[{\hat{a}}_{j},{\hat{b}}_{j}\right]\right\}}_{j=1}^{\hat{K}}$ maximal contiguous predicted alarm segments. A truth event is detected when any predicted segment overlaps it. Event recall is

(25) $$R_{\mathrm{event}}=\frac{1}{K}\sum_{k=1}^{K}I\;\left(\exists j:\left[{\hat{a}}_{j},{\hat{b}}_{j}\right]\cap \left[a_{k},b_{k}\right]\neq ⌀\right).$$

Unlike point adjustment, this definition does not replace all point predictions inside a detected event. The point-wise predictions remain unchanged for AUPRC, AUROC, and alarm segmentation. For MetroPT-3, E is the four company-reported failure intervals rather than maximal runs reconstructed from a sampled binary vector; this preserves event identity across acquisition gaps.

Because a single overlapping alarm can make Equation (25) equal to one, we also quantify how much of each labeled interval is covered. Let $T_{k}$ be the scored timestamps within event *k*, with $N_{k}=\left|T_{k}\right|$. Its alarm-coverage ratio and the macro-average over events are

(26) $$C_{k}=\frac{1}{N_{k}}\sum_{t\in T_{k}}{\hat{y}}_{t},\;C_{\mathrm{event}}=\frac{1}{K}\sum_{k=1}^{K}C_{k}.$$

A missed event therefore contributes zero coverage rather than disappearing from the summary. This is a strict point-overlap fraction, not a point-adjusted or tolerance-expanded range score.

A predicted segment is false when it overlaps no truth event. For scored duration *H* hours,

(27) $$FA/h=\frac{1}{H}\sum_{j=1}^{\hat{K}}I\;\left(\forall k:\left[{\hat{a}}_{j},{\hat{b}}_{j}\right]\cap \left[a_{k},b_{k}\right]=⌀\right).$$

Counting segments rather than isolated false-positive timestamps better represents operator interruptions, although it does not encode the human cost of alarm duration or severity.

For a detected truth event, first-hit delay is

(28) $$\delta_{k}=\min\left\{t-a_{k}:t\in \left[a_{k},b_{k}\right],{\hat{y}}_{t}=1\right\}.$$

Let $\ell_{k}$ denote event duration in the evaluator’s native unit. For regular index vectors, $\ell_{k}=N_{k}$ scored steps; for MetroPT-3, it is the company report’s wall-clock stop time minus start time. The range-normalized delay is ${\tilde{\delta }}_{k}=\delta_{k}/\ell_{k}$. Delay summaries condition on detection and are therefore interpreted together with event recall and Equation (26); missed events have zero coverage and undefined delay. Reporting low delay alone would be misleading if many events were never detected.

For metric *M*, every comparison begins with a paired difference at the admitted unit,

(29) $$\Delta_{s,e}^{\left(M\right)}=M_{s,e}^{\mathrm{RC}\text{-}\mathrm{WMRAD}}-M_{s,e}^{\mathrm{comparator}},$$

where *s* indexes seed and *e* indexes entity or channel when available. For SMD, MSL, and SMAP, one hierarchical bootstrap draw independently resamples five seeds and the dataset’s entities/channels, then averages the corresponding submatrix:

(30) $$\Delta^{*(b)}=\frac{1}{n_{s}n_{e}}\sum_{i=1}^{n_{s}}\sum_{j=1}^{n_{e}}\Delta_{s_{i}^{*(b)},e_{j}^{*(b)}}.$$

The 2.5th and 97.5th percentiles of 20,000 draws form the reported interval. SKAB and PSM use paired-seed resampling because they do not provide an admissible multi-entity matrix. Bootstrap intervals quantify variation over the represented units; they do not correct dataset bias or justify extrapolation to new industrial sites [41,42]. No cross-dataset averaging is performed because seeds, entities, channels, and pooled files are not exchangeable units. No benchmark-wide comparison was designated as a confirmatory hypothesis test. The many dataset–comparator–metric intervals are therefore reported as exploratory, unadjusted effect summaries; we make no multiplicity-controlled “significant” claims. A future confirmatory study would need to preregister a small primary contrast family and its error-control procedure.

## 5. Results

### 5.1. Dataset-Specific Evidence Landscape

**Figure 3:**
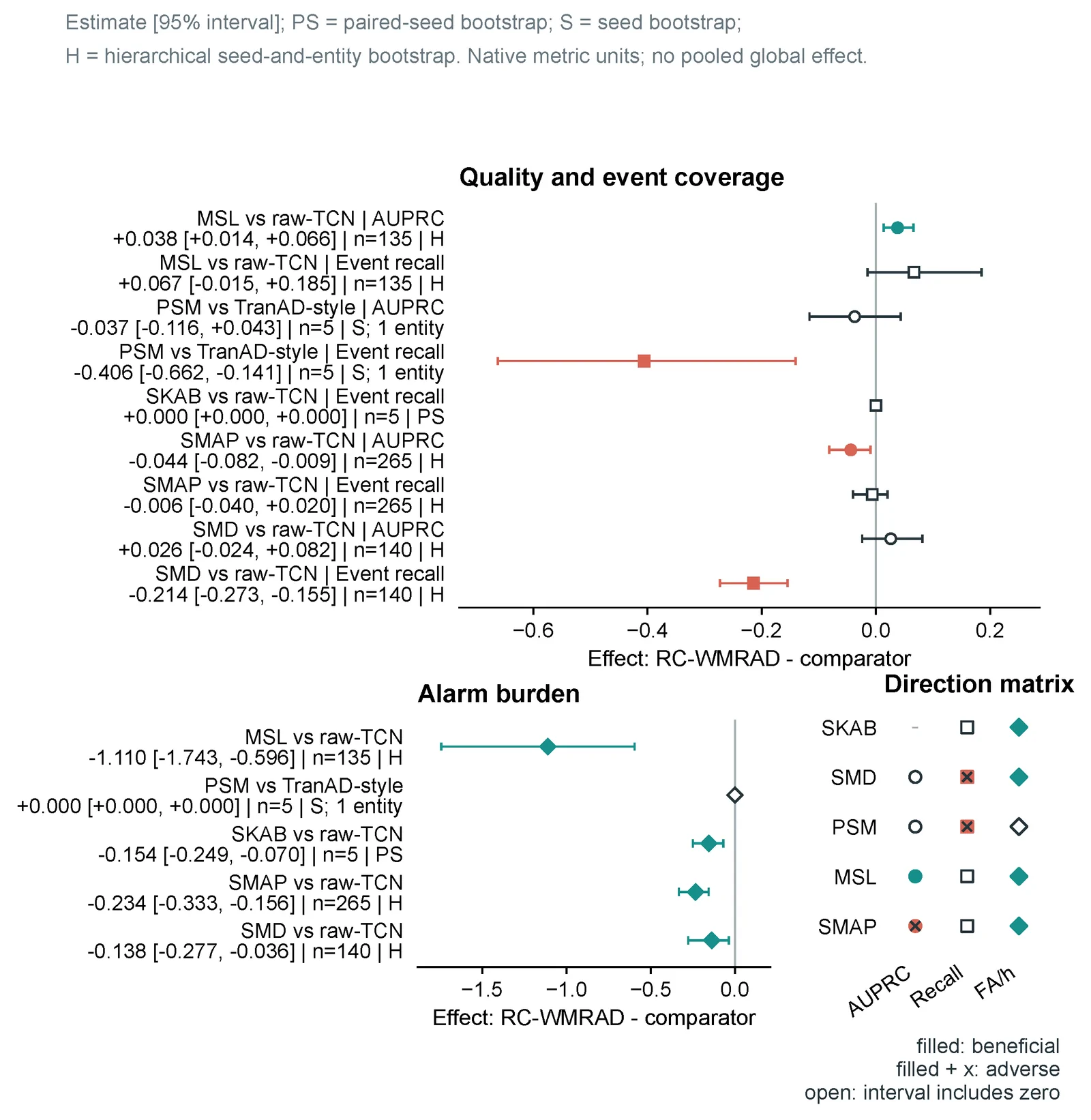
**Cross-dataset evidence landscape.** Effects compare RC-WMRAD with Raw-TCN (PSM: TranAD-style). Intervals respect dataset-specific statistical units; normal-only thresholds are locked and effects are not pooled.

The effects differed by comparator, dataset, and metric (Figure 3). Relative to Raw-TCN, RC-WMRAD reduced false alarms on SKAB, SMD, MSL, and SMAP. This reduction coexisted with lower SMD event recall, higher MSL AUPRC, and lower SMAP AUPRC. PSM versus TranAD-style showed lower event recall and an AUPRC interval spanning zero. Table 3 reports the admitted values without pooling statistical units.

Three observations follow from this landscape. First, the sign of the false-alarm effect was more consistent than the sign of the ranking effect against Raw-TCN. That consistency does not prove a common mechanism because alarm segments depend jointly on score geometry and independently calibrated thresholds. Second, MSL and SMAP demonstrate why a single claim of better spacecraft performance would be invalid: MSL supports an AUPRC gain, whereas SMAP supports an AUPRC loss, even though both show lower alarm burden. Third, changing the comparator changes the question. The PSM TranAD-style result cannot be interpreted as a direct extension of the Raw-TCN comparison because the architectures, score distributions, and operating points differ.

The intervals also have different inferential meanings. SMD, MSL, and SMAP intervals propagate both seed and entity/channel variation through Equation (30); PSM intervals represent seed variation on one entity; and SKAB’s main inferential display resamples paired seeds from a pooled-file evaluator. The visual alignment of these intervals is useful for comparison, but does not make their sampling populations exchangeable.

### 5.2. External Industrial Comparator Breadth

MetroPT-3 is the only experiment in which all five modeling families share one physical asset, the same five seeds, the same first-month normal-development roles, and the same four failure reports (Table 4). Against Raw-TCN, RC-WMRAD reduced false alarms by 0.595 per hour with a paired 95% interval of [−1.068,−0.103], while its AUPRC and event-recall intervals crossed zero. TranAD-style achieved the strongest mean AUPRC and shortest median delay, but produced approximately twice RC-WMRAD’s false-alarm rate; RC-WMRAD-minus-TranAD-style AUPRC was −0.0109[−0.0204,−0.0044] and false alarms per hour amounted to −0.5095[−0.5944,−0.4403].

The additional families change the comparison boundary without reversing the conditional interpretation. RC-WMRAD-minus-USAD-style event recall was +0.150[+0.050,+0.250], while the AUPRC and false-alarm intervals crossed zero. Against GDN-style, the event-recall difference was +0.550[+0.400,+0.700], again with unresolved AUPRC and false-alarm differences. These intervals represent five fits of one asset, not five independent railway systems. The experiment therefore broadens architecture coverage and industrial relevance but cannot establish population-level superiority.

### 5.3. Independently Optimized Component Ablation

The independently optimized SKAB ablation tests whether the clean-condition behavior survives removal or restriction of individual components (Table 5). Unlike the frozen-path event attribution, every A0–A5 row has its own optimization and calibration run for each seed. The entity-level analysis retains 40 profiled SKAB entities per seed, giving 200 paired entity–seed observations before metric-specific undefined values are removed.

The strongest resolved effect concerns alarm burden rather than ranking. Removing the wavelet path in A1 increased the mean entity-level false-alarm rate by 2.992 alarms/hour, with a 95% paired bootstrap interval of [0.995,5.532]. Its AUPRC interval crossed zero. The wavelet-only, fixed-fusion, global-gate, and no-mask-gate variants also had AUPRC intervals crossing zero. Event recall was saturated at 1.0 for all clean SKAB variants, so this dataset cannot identify a clean-condition event-coverage contribution. In particular, A4 and A5 are effectively indistinguishable from A0 when histories and targets are fully observed; their purpose is instead to expose the gate under missingness. These findings support a conditional alarm-burden role for combining the raw and wavelet paths, not universal superiority of every proposed component.

### 5.4. Event Anatomy and Entity Heterogeneity

**Figure 4:**
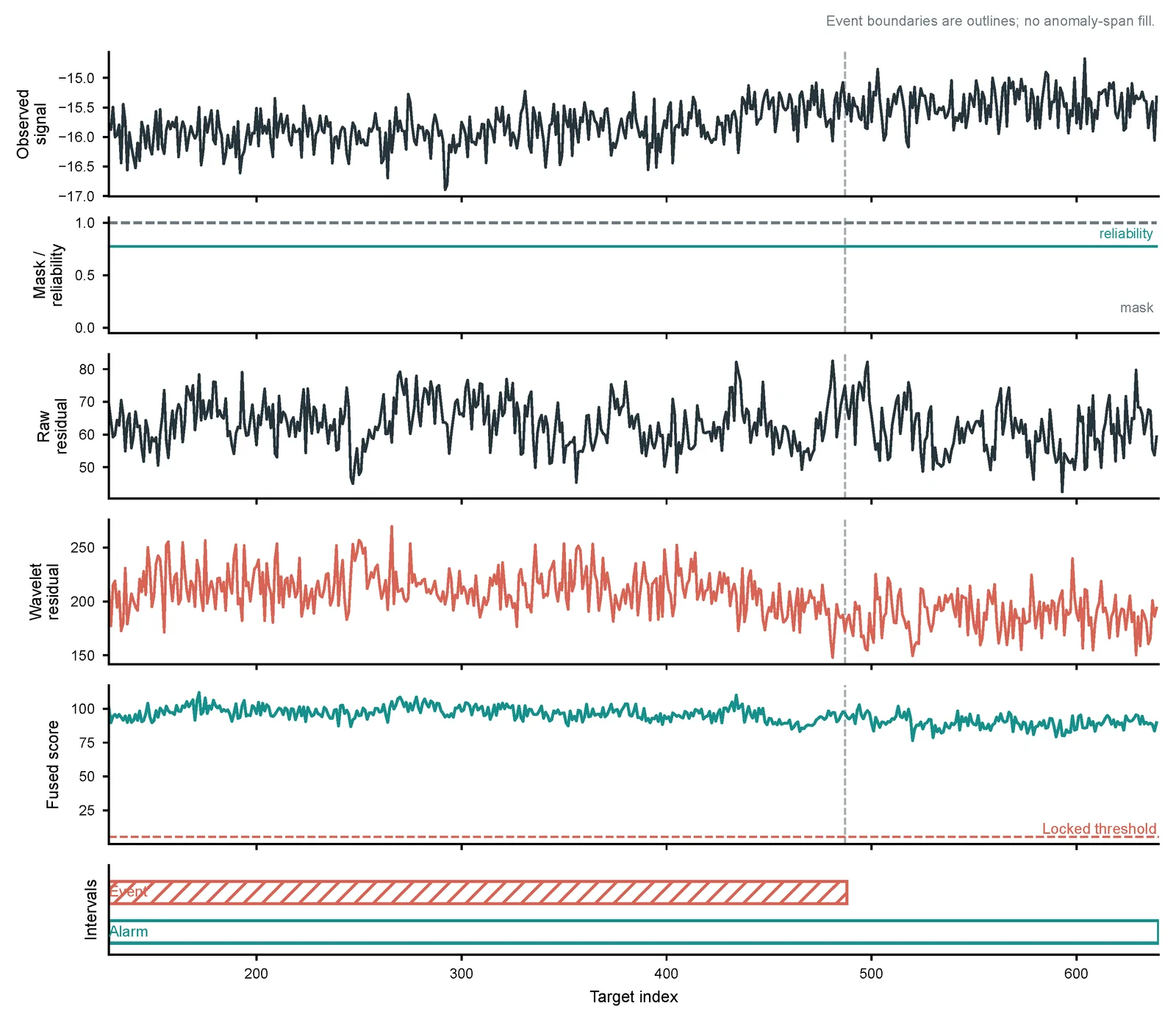
**Event-level multitrack anatomy.** One frozen SKAB window shows observed input, missingness, residual paths, fused score, locked seed-0 threshold, and alarm segments. The case is explanatory rather than comparative and is not representative of all events.

**Figure 5:**
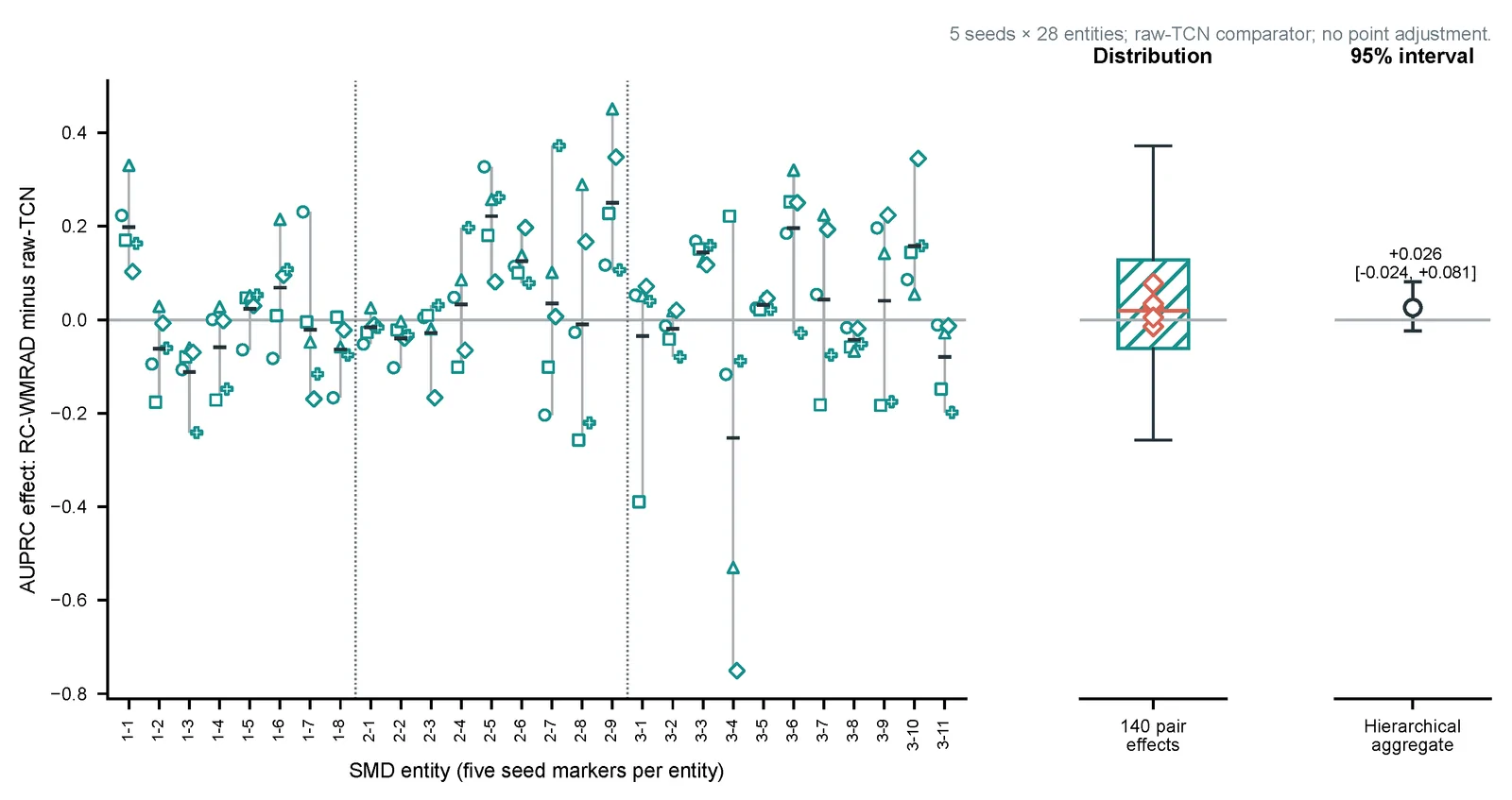
**SMD entity-level heterogeneity.** Clean AUPRC effects compare RC-WMRAD with Raw-TCN across five seeds and 28 entities. Circle, square, triangle, plus, and diamond markers denote the five individual seeds within each entity. The aggregate uses a 95% hierarchical seed–entity bootstrap interval; the 140 paired rows are not independent replicates.

The deterministic SKAB trace connects observed input, missingness, residual paths, fused score, threshold, and alarm segments at one target index (Figure 4). It explains score formation but carries no comparative estimate. The SMD distribution then exposes variation hidden by the aggregate AUPRC effect (Figure 5). Across 140 clean seed–entity pairs, the mean AUPRC effect was positive but its hierarchical interval included zero, while individual entity effects varied substantially. The hierarchical interval treats seeds and entities as distinct resampling levels.

The trace illustrates an important ordering detail. Raw and wavelet predictions are generated from the history; only after the target arrives are Equations (16) and (17) evaluated. The target label is still unavailable at this stage. The fused score is then compared with a threshold already fixed by Equation (22). Consequently, the plotted event shading explains evaluation context but does not participate in score construction.

SMD heterogeneity cautions against treating 140 rows as 140 independent experiments. Rows sharing a seed inherit the same initialization and training configuration, while rows sharing an entity inherit the same physical series and anomaly annotation. The hierarchical bootstrap preserves these two sources of repeated structure. Entity-wise sign changes further indicate that an aggregate mean cannot identify which sensor systems benefit. A future deployment decision would require entity characteristics, failure types, and operating costs that are not encoded by the benchmark identifier alone.

### 5.5. Comparator Trade-Offs and Event Attribution

**Figure 6:**
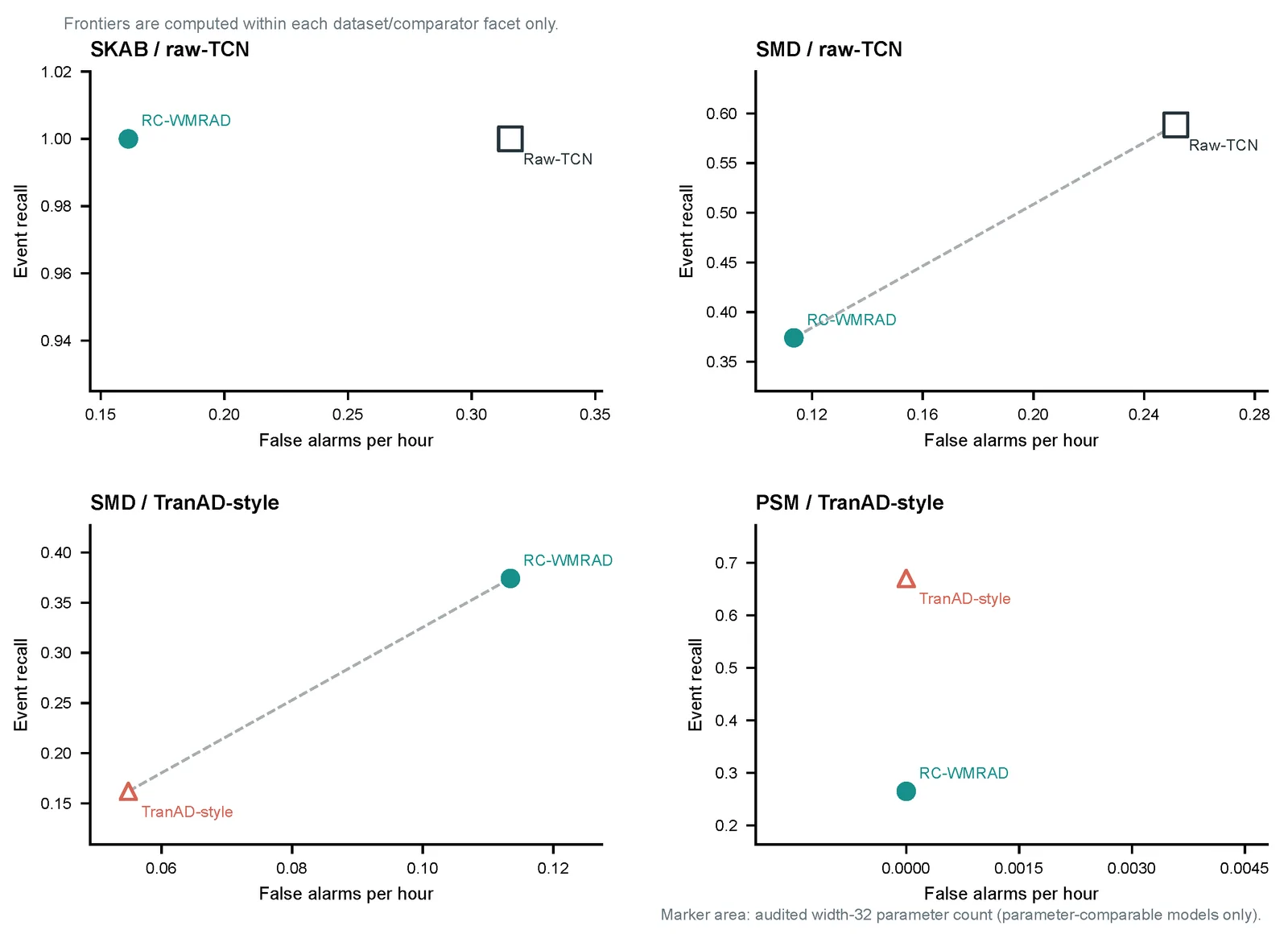
**Dataset-faceted Pareto trade-off.** Matched operating-point means compare RC-WMRAD with Raw-TCN or TranAD-style within each facet. Normal-only thresholds are locked independently; points are not pooled and within-facet frontiers do not imply cross-dataset dominance.

**Figure 7:**
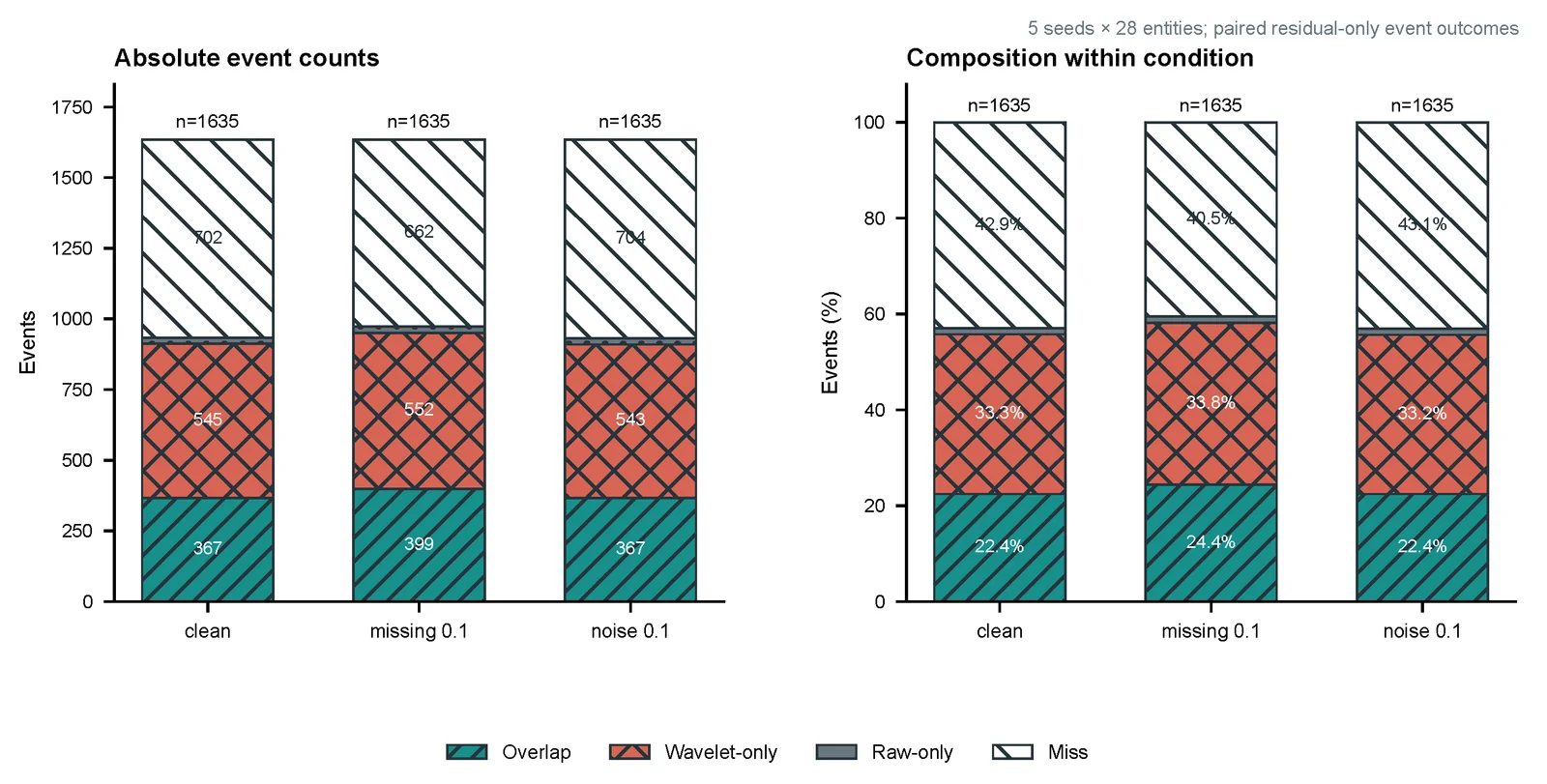
**Event-coverage attribution.** Learned fusion is compared with fixed raw-only and wavelet-only rescoring over 1635 paired SMD events per condition. Counts are exhaustive under independently locked thresholds; the post-primary analysis is diagnostic, not a ranking of deployable models.

Faceting by dataset and comparator prevents unlike operating points from forming a single frontier (Figure 6). RC-WMRAD occupies different ranking, recall, and alarm-burden positions against Raw-TCN and TranAD-style. The result is conditional deployment evidence, not dominance. Event attribution clarifies one SMD boundary (Figure 7). On clean data, learned fusion and the raw-only path detected 388 events together, learned fusion alone detected 125, and neither detected 1122. Against wavelet-only scoring, 488 were detected by both paths, 424 by wavelet-only, 25 by learned fusion, and 698 by neither. These counts localize event-coverage differences without changing the primary comparison.

A point is Pareto-dominated within a facet only when another operating point is no worse on every displayed objective and strictly better on at least one. The facets intentionally prevent a low false-alarm value from one dataset being compared numerically with an AUPRC value from another. They also prevent the TranAD-style comparison from being folded into the Raw-TCN frontier. This presentation exposes trade-offs but does not choose an operating utility for the user.

The event-attribution counts are based on independently calibrated fixed paths. This condition matters: applying the learned-fusion threshold to raw-only or wavelet-only residuals would confound representation with score scale. Wavelet-only coverage of 424 events missed by learned fusion suggests that potentially useful band evidence is suppressed at the selected operating points. However, the 698 events missed by both paths show that branch selection alone cannot resolve most undetected events. Furthermore, a path that covers more events may produce a different false-alarm stream. The attribution is therefore diagnostic evidence about coverage, not a recommendation to replace the learned detector.

### 5.6. Robustness and Calibration Boundaries

**Figure 8:**
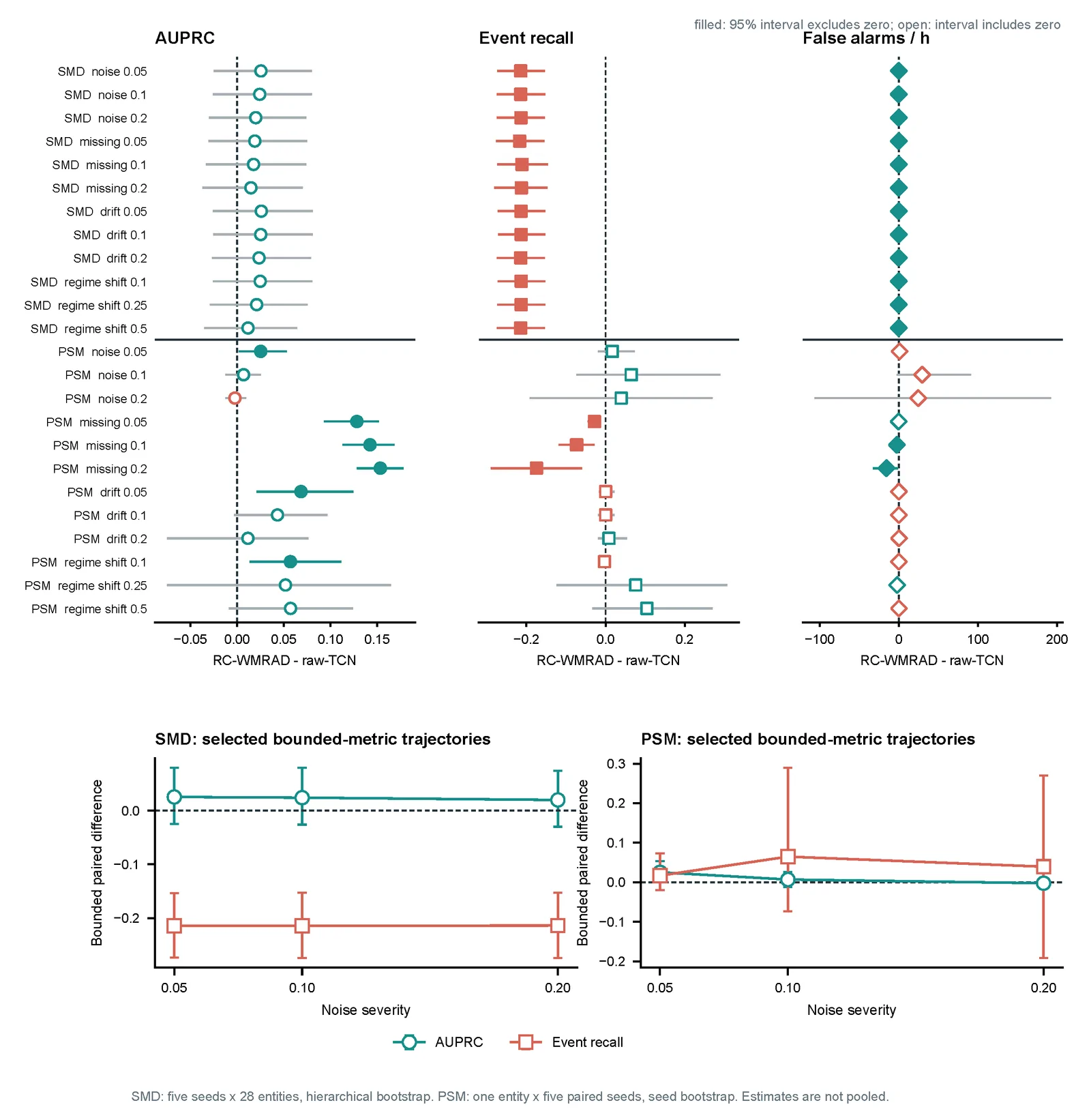
**Robustness landscape.** RC-WMRAD-minus-Raw-TCN effects use hierarchical seed–entity intervals for SMD and paired-seed intervals for the single PSM entity. Clean normal-only thresholds remain locked across severities; datasets are shown separately and not pooled.

Corruption effects remained dataset- and metric-specific (Figure 8; Table 6). Across all twelve SMD severity conditions, AUPRC intervals included zero, event recall was lower, and false alarms were lower. PSM missingness produced higher AUPRC and lower event recall at all three severities, while noise and regime-shift effects were less stable. No cross-dataset averaging is used, and these grids establish no universal robustness.

The formal corruption grid is restricted to SMD and PSM because only those two benchmarks have the complete paired five-seed chain required for this analysis: clean and corrupted score bundles, locked clean normal-only thresholds, source hashes, and a common evaluator for all four corruption families. Extending the table to datasets with incomplete or differently generated artifacts would mix evidence standards. This restriction preserves comparability but narrows the robustness claim; it is not evidence that the two datasets represent the full deployment domain.

The SMD pattern is internally consistent but not uniformly beneficial. Lower event recall and lower false alarms can both arise when a locked score distribution becomes more conservative relative to its threshold. AUPRC, which integrates ranking over thresholds, does not support a systematic gain across the grid. This combination argues against describing the result as robust improvement. It instead identifies a stable sensitivity–burden shift under the tested corruption generator.

PSM displays a different boundary. Under missingness, point ranking improves while event recall decreases. This is possible because AUPRC summarizes ordering over all candidate thresholds, whereas Equation (25) evaluates one threshold fixed from clean normal calibration. Improved global ranking therefore need not place enough samples from every event above the selected operating point. Noise conditions with wide false-alarm intervals additionally show that five seeds on one entity provide limited resolution for unstable alarm counts.

**Figure 9:**
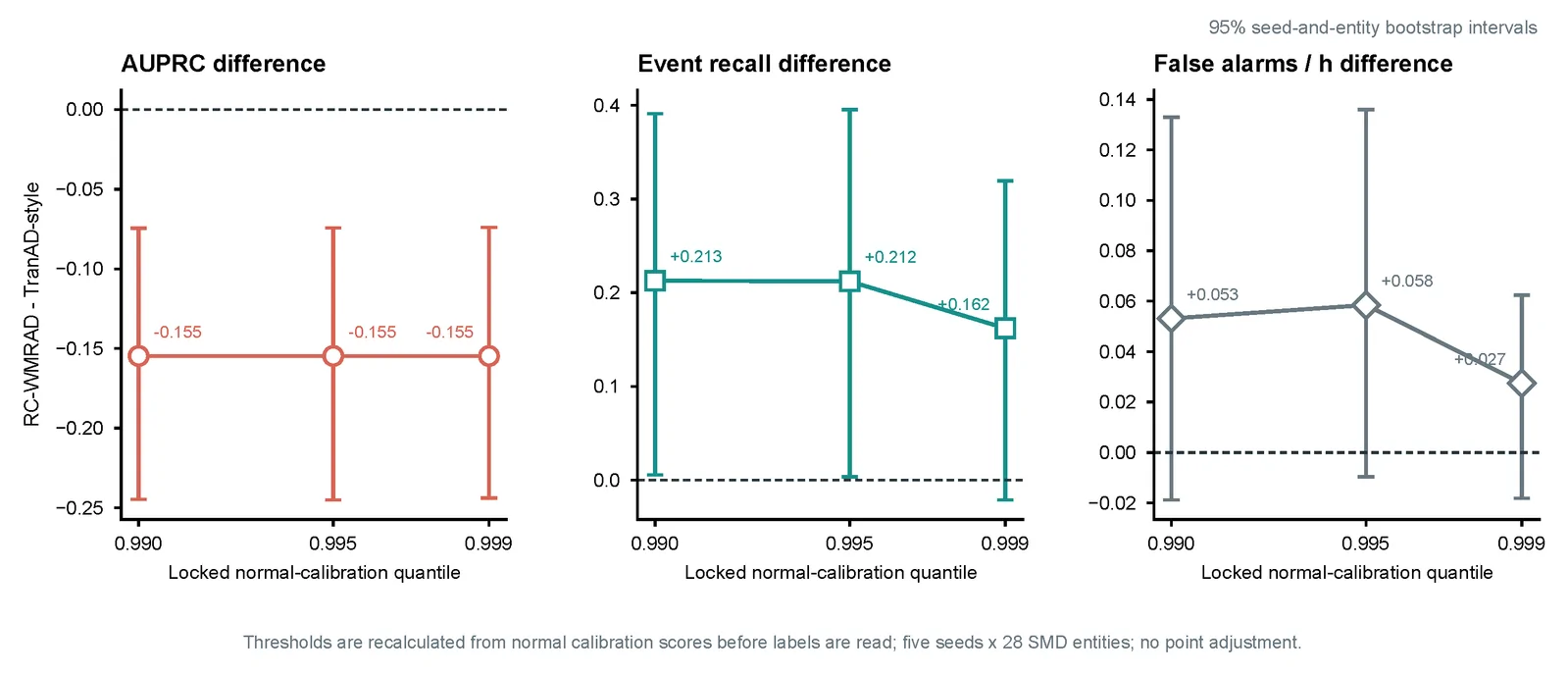
**Calibration sensitivity.** SMD effects versus TranAD-style use 140 paired seed–entity observations and 95% hierarchical intervals. Thresholds are recalculated only from locked normal scores; three nearby quantiles test local sensitivity rather than unrestricted optimization.

The SMD comparison against TranAD-style was then recalibrated at normal-score quantiles 0.990, 0.995, and 0.999 (Figure 9). The AUPRC difference remained −0.1547 at all three quantiles. Event-recall differences were positive at 0.990 and 0.995 but became uncertain at 0.999. All false-alarm intervals included zero. The ranking boundary is stable over this narrow range, whereas event coverage depends on the operating point.

AUPRC is unchanged because recalibration does not alter score ordering. Event recall and false alarms change because the quantile controls how much of each normal-calibration tail is admitted. The sensitivity analysis is intentionally local: three predeclared quantiles cannot establish threshold optimality, and choosing among them using test labels would violate the protocol. Its purpose is to show which conclusions survive a plausible nearby change in normal-only calibration.

MetroPT-3 additionally tests calibration sample size without changing any trained model or test score (Table 7). Chronological prefixes contain 532, 1332, 2664, or 5329 normal scores, corresponding to 10%, 25%, 50%, and 100% of the calibration stream. RC-WMRAD event recall remains 0.95 at every size, but 10% calibration increases false alarms by 0.236/hour relative to the full prefix, with a paired 95% seed interval of [0.094,0.363]. The 25% difference is similarly positive, whereas the 50% interval crosses zero. Raw-TCN and TranAD-style are non-monotone: more calibration data does not necessarily move an empirical upper quantile in one direction. AUPRC and AUROC remain exactly invariant because the frozen score ordering is unchanged.

### 5.7. Operational Burden and Complexity Audit

**Figure 10:**
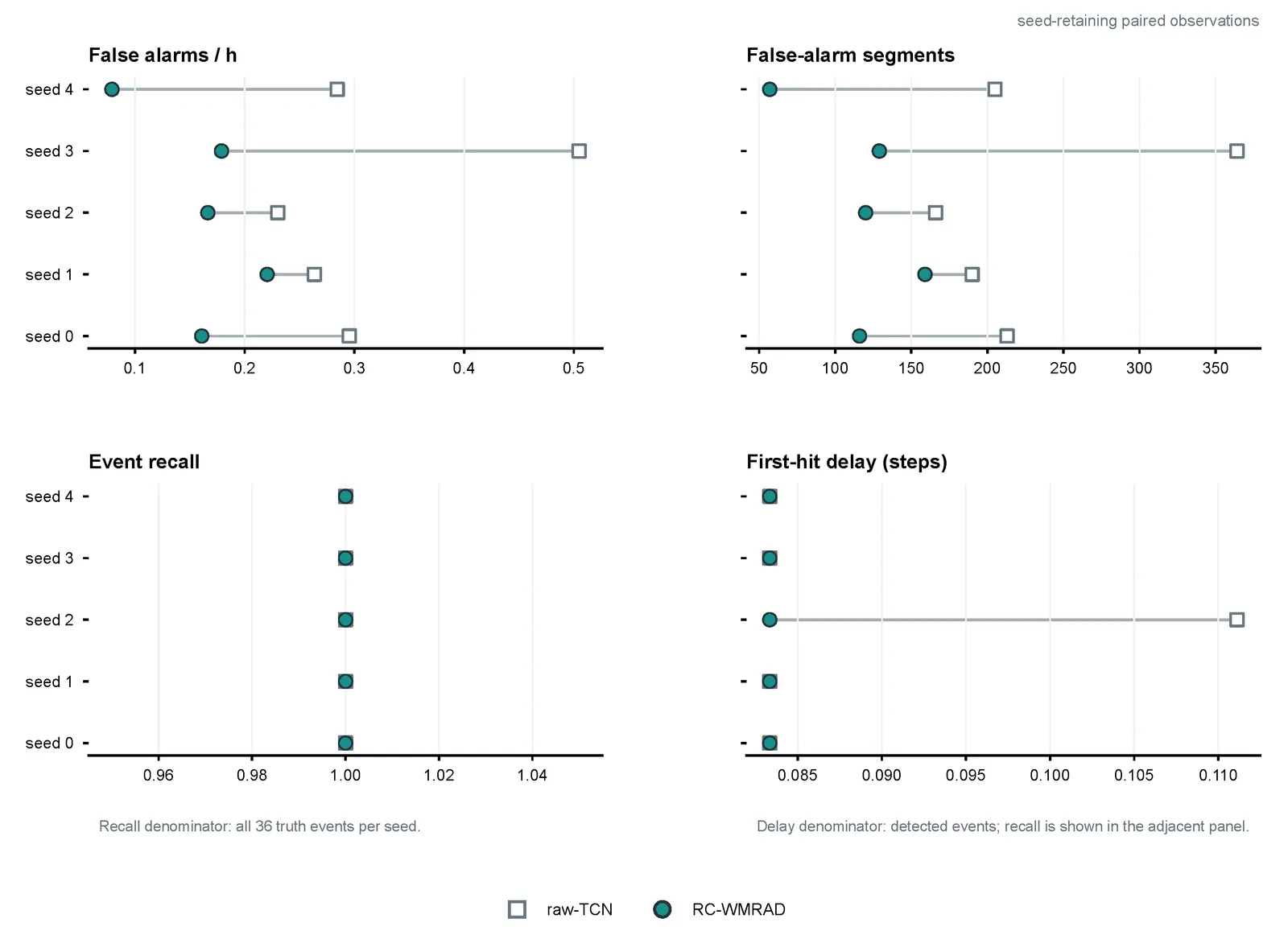
**Alarm burden and detection delay.** Five matched SKAB seeds compare RC-WMRAD with Raw-TCN under independently locked thresholds. Paired observations are retained directly; delay is conditional on detection and pooled files do not provide entity-level uncertainty.

Seed-retaining SKAB evidence separated alarm burden from delay (Figure 10). Event recall was 1.0 for both methods in every seed. Mean false alarms per hour were 0.1611 for RC-WMRAD and 0.3156 for Raw-TCN. First-hit delay differed by −0.0056 samples on average, with delay defined only over detected truth events. The operational result is therefore lower alarm segmentation under this pooled-file evaluator, not a broad accuracy gain.

Because every truth event is detected in this comparison, the delay denominator is identical for the two models and the near-zero delay difference is directly interpretable. The false-alarm result indicates fewer non-overlapping alarm segments, not necessarily fewer false-positive timestamps or shorter alarm duration. The pooled SKAB evaluator also prevents entity-level uncertainty estimation. These restrictions are why the result is phrased as an operational alarm-segmentation effect rather than as a universal detection improvement.

**Figure 11:**
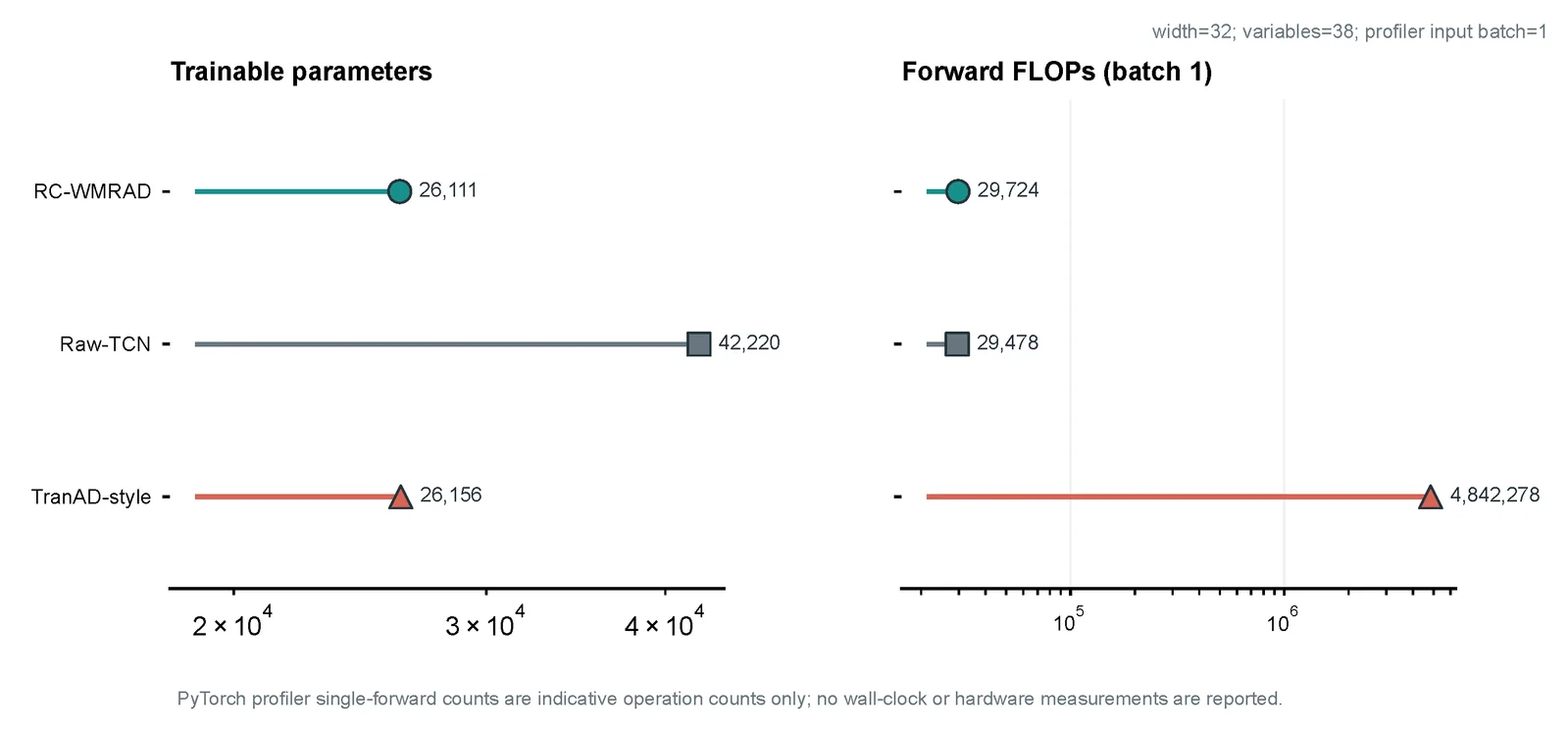
**Complexity audit.** Deterministic parameter and batch-one forward-FLOP counts compare width-32 RC-WMRAD, Raw-TCN, and TranAD-style models. These architecture counts are not measurements of runtime; measured latency is reported separately in Table 9.

The width-32 complexity audit (Figure 11; Table 8) reports 26,111 trainable parameters and 29,724 batch-one forward FLOPs for RC-WMRAD. Raw-TCN has 42,220 parameters and 29,478 forward FLOPs, while TranAD-style has 26,156 parameters and 4,842,278 forward FLOPs. These remain architecture and profiler counts, so the complexity audit alone supports no runtime claim. A separate measured audit on an Intel Core i7-12700H CPU and NVIDIA RTX 3060 Laptop GPU provides hardware-specific batch-one latency (Table 9). It uses PyTorch 2.10, one CPU thread, 200 warm-up calls, and 1000 synchronized measurements.

Formally, the trainable parameter count is

(31) $$P\left(\theta \right)=\sum_{\ell \in L_{\mathrm{train}}}\prod_{d\in \mathrm{shape}(\theta_{\ell })}d,$$

and the reported FLOP value is the profiler’s sum of primitive operation counts for one forward call at the audited input shape,

(32) $$F_{\mathrm{forward}}=\sum_{o\in G_{\mathrm{forward}}}F\left(o\right).$$

Neither quantity includes optimizer state, backward computation, data transfer, kernel-launch overhead, or hardware utilization. Similar FLOPs can have different wall-clock cost, while similar parameter counts can have very different attention or convolution workloads. RC-WMRAD and Raw-TCN are therefore forward-compute-near controls under this profiler; TranAD-style is a parameter-near but compute-different comparator.

At batch size one, RC-WMRAD’s CPU model-only median was 1.325 ms (approximately 723 calls/s), while its synchronized GPU median was 2.603 ms. The GPU result is slower because launch, synchronization, and small-kernel overhead dominate this small model and batch; it is not evidence that the GPU is slower at larger batches. RC-WMRAD used a 106.8 kB serialized state dictionary and reached 8.5 MB peak allocated GPU memory in the audit process. Its CPU p50 increased from 1.155 ms at L=32 to 1.474 ms at L=256. These measurements support millisecond-scale warm inference on the named workstation only.

### 5.8. Industrial Deployment Scenario

A practical RC-WMRAD installation can be expressed as a bounded monitoring chain rather than as a stand-alone classifier. A timestamped value–mask packet first enters the causal forward-fill and frozen median/MAD transform. The service retains the latest 128 samples, approximately 21 min at MetroPT-3’s median 10-s spacing, and produces one decision per minute. The resulting score is compared with the pre-installed normal-only $Q_{0.995}$ threshold. Consecutive positive decisions are merged into one alarm segment and routed with the triggering interval, available sensor values, and threshold provenance to an operator or maintenance system. This alarm interface is a proposed systems mapping of the evaluated score contract; no operator study or live maintenance integration was performed.

**Figure 12:**
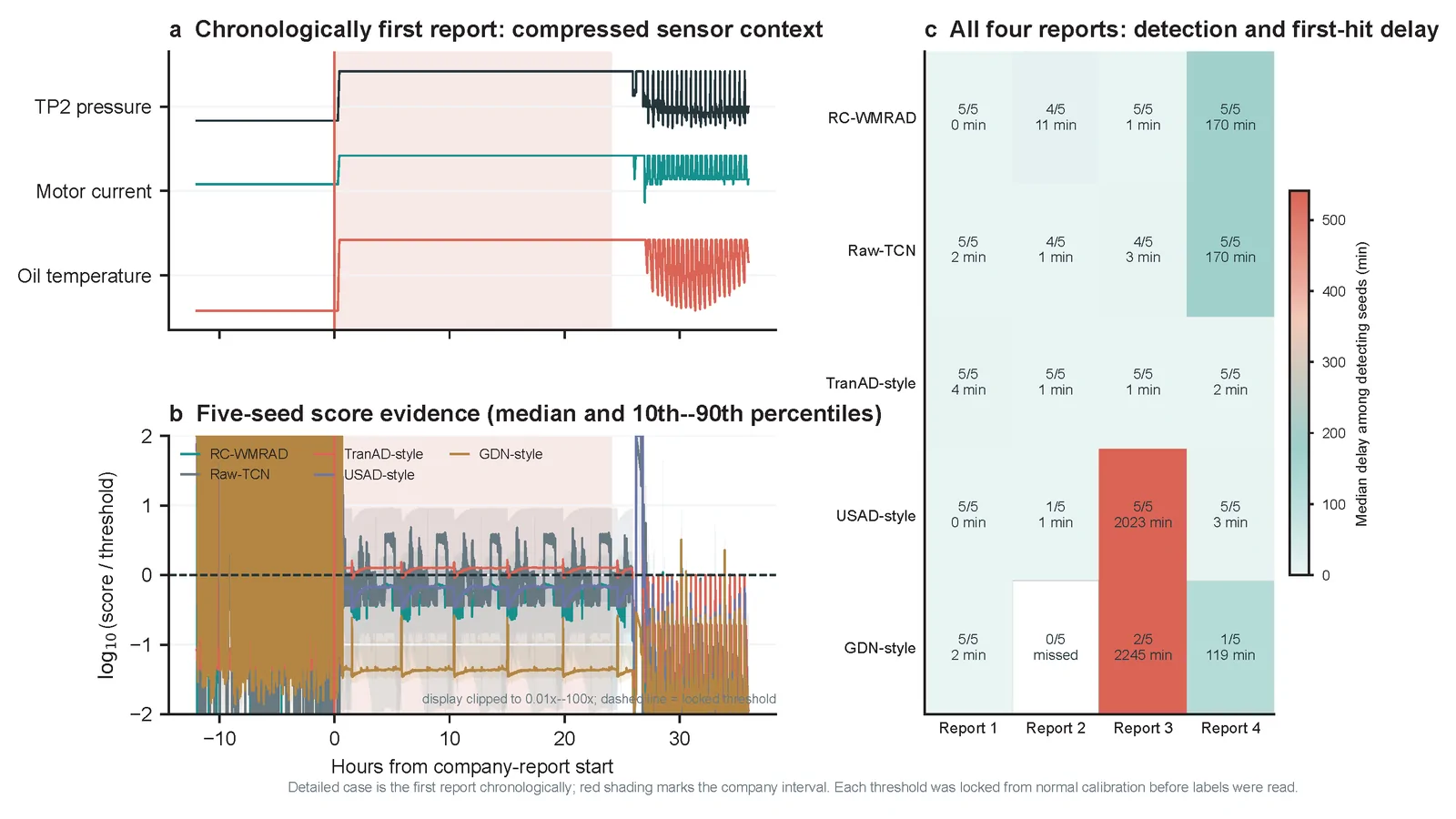
**MetroPT-3 industrial failure case.** The first company report chronologically is shown in detail; all four reports and five matched seeds remain in the matrix. Scores are relative to post-lock normal-only thresholds, labels are used only for visualization and evaluation, and no point adjustment is applied.

Figure 12 provides a post-lock retrospective example. The detailed window is the first company report chronologically, not the event with the most favorable model outcome. Sensor tracks are compressed only for display, while every model trace is divided by its own seed-specific locked threshold. The right panel retains all four reports and all five matched seeds. TranAD-style detected every report in every seed with median first-hit delays no greater than 4.1 min. RC-WMRAD detected 19 of 20 seed–report pairs, but its Report 4 median first hit was 170.35 min after onset, or 63.1% of that report’s duration; its median Report 4 coverage was only 33.0%. This is inadequate as early warning and rules out using the present RC-WMRAD operating point as the sole monitor for that failure pattern. GDN-style missed Report 2 in all seeds and detected Report 4 in one seed; USAD-style detected Report 2 in one seed and had a 2023-min median delay on Report 3. These cases expose failure-specific operating behavior and do not replace the aggregate ranking and alarm-burden comparisons. Table 10 reports the corresponding partial-event coverage and normalized delay for every model.

Table 11 separates components already exercised by the retrospective protocol from requirements that remain untested. We additionally ran a locked workstation systems audit using the MetroPT-3 seed-0 checkpoints and real, label-free MetroPT-3 CSV ingestion. Ten fresh-process RC-WMRAD starts required 3033.5 ms at the median (3258.2 ms at p95). In 30-s rolling-history trials, RC-WMRAD sustained 335.2 requests/s on CPU and 179.5 requests/s on CUDA; synchronized inference latency was 1.772 [3.065] ms on CPU and 3.314 [3.949] ms on CUDA (p50 [p95]). The small model was faster on CPU in this batch-one protocol because CUDA transfer and synchronization overhead dominated. CUDA throughput increased from 347.7 requests/s with one worker to 441.1 with two, but did not improve with four workers (429.4 requests/s), whose p95 latency rose to 10.747 ms. Three cached 4096-row CSV reads sustained 197,864 rows/s, and 200-ms telemetry during the CUDA run recorded a maximum temperature of 64 °C and maximum board power of 30.62 W. These short workstation measurements are not an energy-efficiency, long-duration thermal, embedded-target, or field-deployment qualification. A field implementation would additionally need timestamp-quality monitoring, alarm persistence and acknowledgment rules, secure state/version logging, and a prospective normal-data recalibration trigger defined before failure outcomes are available.

## 6. Discussion

### 6.1. A Conditional Claim–Evidence Chain

The combined evidence changes the interpretation from a single model ranking to a set of operating trade-offs. Lower alarm burden appears on SKAB, SMD, MSL, and SMAP, but its companion effects differ. On SMD, fewer false alarms are accompanied by lower event coverage against Raw-TCN. On MSL, the proposed score improves AUPRC and lowers alarm burden, while its event-recall interval includes zero. On SMAP, the lower alarm burden accompanies lower AUPRC and uncertain event-recall change. PSM versus TranAD-style does not reproduce the SMD event-coverage advantage. The study therefore makes no universal superiority claim, estimates no pooled global effect, and performs no cross-dataset averaging.

This separation is necessary because the reported metrics answer distinct mathematical questions. AUPRC summarizes the ordering induced by the continuous score $s_{t}$ over all possible thresholds and is especially informative under class imbalance [1,3]. Event recall evaluates whether the fixed operating point intersects each labeled interval, whereas false alarms per hour quantifies the segmentation burden outside those intervals. A detector can rank points less effectively while crossing a locked threshold on more truth events, or reduce alarm fragmentation while missing events. Reporting all three quantities prevents a favorable rank statistic from substituting for the deployment decision and prevents a low alarm count from being interpreted without its event-coverage cost.

The inferential unit also changes the strength of a claim. Hierarchical seed–entity resampling on SMD represents variation across both stochastic training and machines, but paired-seed intervals on PSM represent only repeated fits to one evaluated entity. Pooled-file SKAB analysis retains paired seeds but cannot estimate between-entity heterogeneity. These designs are not interchangeable, even when their point estimates use the same units. Consequently, the manuscript reports dataset-specific effects rather than treating benchmark identity as a nuisance variable that can be averaged away. This follows the broader warning that time-series anomaly-detection conclusions can be strongly shaped by benchmark construction, labels, and evaluation procedure [5,40].

### 6.2. Wavelet Evidence, Fusion, and Robustness

The fixed-path attribution shows that wavelet-only scores recover events suppressed by learned fusion under independently calibrated thresholds. The result is consistent with the multiresolution view that a local discontinuity can remain visible in a detail coefficient even when it is weak relative to the raw-channel baseline [7,8]. In that SMD attribution, however, the wavelet path is not an independent detector: it shares the causal encoder and the frozen trained checkpoint with the raw path. The attribution therefore identifies where score composition changes event coverage, but it does not prove a causal physical mechanism or recommend wavelet-only deployment. The independently optimized SKAB ablation addresses the narrower architecture question and finds an alarm-burden effect for removing the wavelet path, while leaving most clean-condition ranking intervals unresolved.

The mask-conditioned gate should likewise be interpreted narrowly. Its support term measures observed availability in the input window, not the epistemic correctness of either predictor. The gate can down-weight a branch when recent variables are absent, but it cannot know whether a frequency component has become spurious under drift or whether an unseen regime invalidates the learned residual distribution. The noise and drift results are therefore empirical stress tests of the complete detector, not variables inferred by the gate. This is distinct from parameter- or prediction-uncertainty models [38] and from uncertainty-aware threshold adaptation [20]. The contribution here is a deterministic availability-conditioned fusion rule whose inputs and label isolation can be audited.

The severity grids further constrain interpretation. SMD preserves a lower-alarm and lower-recall pattern across the tested families, whereas PSM contains narrow benefits and unstable regions. Missingness improves PSM AUPRC while reducing recall, and high-noise alarm effects are uncertain. Synthetic noise, missingness, drift, and regime shifts are controlled probes; they do not reproduce all sensor faults, maintenance interventions, changing loads, or correlated failures encountered in operation. The results therefore make no universal robustness claim and do not extrapolate beyond the tested severities.

Calibration sensitivity clarifies another boundary. Changing the normal-score quantile alters τ and hence the binary alarm sequence, but it cannot change AUPRC because the ordering of $s_{t}$ is fixed. The stable negative SMD AUPRC difference against TranAD-style is thus a ranking result, while the event-recall change across quantiles is an operating-point result. This distinction also explains why post hoc calibration can improve threshold behavior without repairing a deficient score ranking [18,19]. A deployment study would need prospective recalibration schedules and drift alarms rather than selecting a favorable quantile retrospectively.

### 6.3. Operational and Computational Boundaries

Alarm segmentation, interval coverage, and delay are necessary companions. False alarms per hour approximates operator interruption frequency when each contiguous positive segment creates one review task. It does not measure alarm duration, the number of anomalous timestamps inside a false segment, or the cognitive cost of resolving an alert. First-hit delay has the opposite conditioning problem: it is defined only for detected truth events. A low mean delay can therefore coexist with poor event recall if difficult events are omitted from the denominator, while one late hit can still yield full event recall despite negligible coverage. The MetroPT-3 coverage audit exposes both cases, including RC-WMRAD’s operationally unacceptable Report 4 delay. The SKAB result likewise avoids a delay-only interpretation by displaying recall, segmentation burden, and delay together. Its pooled-file unit nevertheless limits uncertainty claims, and entity-resolved prospective logging would provide a stronger operational estimate.

The complexity comparison is similarly bounded. Parameter parity does not imply forward-compute parity, and FLOP counts did not predict the measured batch-one ordering. Convolution shape, attention kernels, memory traffic, numerical precision, kernel launches, and transfer overhead all contribute to wall-clock behavior. Beyond the warm batch-one comparison, the locked systems audit now measures fresh-process cold start, 30-s CPU and CUDA rolling-history operation, one-/two-/four-worker concurrency, bounded real-CSV ingestion, and 200-ms temperature and power telemetry on the named workstation and software stack. The throughput maximum at two workers and the higher four-worker tail latency show that concurrency cannot be inferred from single-call latency. The short telemetry interval measures workstation board power and temperature, not task energy, sustained thermal equilibrium, or embedded efficiency.

Taken together, the evidence supports a practical decision rule rather than a universal leaderboard statement. RC-WMRAD is most defensible when a user values an auditable normal-only threshold, causal preprocessing, an explicit alarm-burden analysis, and millisecond-scale warm inference on workstation-class hardware, and can tolerate benchmark-dependent changes in ranking or event coverage. It is not justified when the application requires guaranteed event recall, broad robustness outside the tested corruptions, or certified embedded real-time execution. Making these exclusion conditions explicit is part of the contribution because it connects each claim to the artifact and statistical unit that can actually support it.

The MetroPT-3 case and deployment-readiness matrix make the corresponding systems boundary concrete. The present implementation can supply timestamp-aligned scores, threshold-relative evidence, and alarm segments at a cadence far slower than measured workstation inference. It cannot yet supply a validated operator workflow, maintenance benefit, prospective adaptation policy, or certified edge execution. Cross-domain clinical HCI studies show why this interface layer requires separate evaluation: model and human errors can be complementary, and supervisory review tools can introduce both workflow benefits and over-skimming or missing-data risks [49,50]. These studies concern structured clinical-text extraction rather than sensor anomaly detection, so they motivate human-in-the-loop validation requirements but do not validate the proposed industrial workflow. Those missing interfaces define the next field-study protocol rather than hidden assumptions of the current benchmark result.

## 7. Limitations and Future Work

First, the wavelet path is a causal, undecimated Haar surrogate rather than a general stationary wavelet transform, wavelet packet decomposition, or learned filter bank. Haar filters make support and causality transparent, but their piecewise-constant basis may be inefficient for oscillatory or slowly varying signals. Comparing wavelet families, dilation schedules, and learned lifting schemes under an unchanged score-lock protocol would test whether the observed effects arise from multi-scale locality or from this particular basis. Such extensions should report both additional capacity and frequency response so that a flexible transform is not mistaken for a cost-free replacement.

Second, the observation-support gate is based on mask availability and does not encode noise, drift, predictive uncertainty, or band stability. It is therefore possible for both branch predictions to be wrong while receiving high support. The positive scale heads are optimized inside the standardized absolute-residual objective without an explicit likelihood log-scale term; they are operational score normalizers, not identifiable variance estimates. Future work should compare a proper likelihood or constrained-scale objective and could estimate reliability from normal-only residual dispersion, conformal nonconformity, or ensemble disagreement, provided that adaptation remains isolated from evaluation labels. Any learned gate should be compared with the present deterministic rule and with raw-only and wavelet-only paths, because additional gating capacity can otherwise conceal where gains originate.

Third, the statistical evidence is uneven across datasets. SMD supports hierarchical entity evidence, whereas PSM contributes a single evaluated entity and SKAB uses pooled cleaned files. MSL and SMAP have multiple channels but are not treated as independent entities in the same sense as SMD machines. Five seeds characterize stochastic fitting only coarsely, and benchmark labels may contain ambiguous boundaries. Future studies should retain per-asset identities, increase independently trained seeds when feasible, and report sensitivity to event-boundary tolerance using range-aware metrics [4,6].

Fourth, comparator coverage differs by dataset and does not constitute an exhaustive state-of-the-art tournament. Raw-TCN isolates multi-scale residual evidence under a related causal architecture, while TranAD-style, USAD-style, and GDN-style probe Transformer, reconstruction, and graph-predictive families. Only MetroPT-3 includes all five families, and the clean-room “-style” implementations are protocol controls rather than exact upstream reproductions. A broader benchmark should pin verified public implementations, equal preprocessing access, matched hyperparameter budgets, and the same normal-only calibration contract. Results obtained from incompatible label access or point adjustment should not be pooled with the present estimates.

Fifth, the robustness grid covers four synthetic corruption families at three severities on SMD and PSM only, and calibration sensitivity covers three nearby quantiles. These were the datasets with a complete paired five-seed corruption, score-lock, and common-evaluator artifact chain; the restricted scope is an audit constraint, not a claim of domain representativeness. Neither analysis spans every deployment shift. Corruptions are injected according to known recipes and may be easier to characterize than naturally occurring sensor aging, topology changes, or maintenance resets. Future work should add the same complete chain for the remaining benchmarks, chronologically separated domains, and prospective recalibration, with the trigger defined before outcome labels are available. The SMD event-attribution paths remain diagnostic rescoring modes; the separately trained SKAB variants are architecture ablations rather than deployment-ready products.

Finally, the local audit now covers warm model-only and preprocessing-plus-inference calls, bounded CSV ingestion, fresh-process cold start, short sustained rolling-history trials, concurrent workers, and short GPU temperature/power telemetry. It still omits alert persistence and routing, network and storage back-pressure, cold-cache disk ingestion, long-duration thermal equilibrium, task-level energy, concurrent non-model services, and an embedded edge target. It therefore cannot support an energy-efficiency, certified real-time, or field-readiness claim. A systems extension should add quantization and worst-case latency on the intended deployment hardware, long-duration and cold-cache trials, and the complete alert path while preserving numerical tolerance and publishing raw timing traces.

## 8. Reproducibility and Audit Trail

Reported values are derived from tracked run summaries, audit artifacts, and figure manifests. The workflow records preprocessing hashes, calibration-score hashes, threshold manifests, score-bundle hashes, label-read counters, source paths, model configuration, and statistical units. A threshold is accepted only when its manifest points to normal-only calibration scores and the test-label read count remains zero before the score lock. This makes the temporal ordering of calibration and evaluation inspectable rather than relying on a narrative statement.

Figure manifests link plotted values to tabular sources and preserve editable vector outputs. Comparator directions are encoded explicitly so that a favorable sign is not inferred from color. Hierarchical and paired resampling use fixed seeds and record the unit sampled at each stage; 95% intervals are descriptive, exploratory uncertainty summaries and are not multiplicity-adjusted. No benchmark-wide result is presented as a confirmatory significance test. Incomplete evidence is excluded rather than imputed or silently repaired. These controls reduce accidental inconsistency between code, tables, and prose, although they cannot remove errors already present in public labels or source datasets.

The public-data statement identifies the benchmark families used in the study, while repository-local artifacts provide the derived evidence needed to reconstruct the reported comparisons. Reproduction still depends on compatible software versions and access to the original benchmark distributions. For that reason, the audit trail treats hashes, ordered variable names, preprocessing parameters, and model shapes as part of the experimental result rather than incidental implementation detail.

## 9. Conclusions

RC-WMRAD combines causal raw and multi-scale Haar residual evidence, mask-conditioned fusion, normal-only calibration, and a locked label-evaluation protocol. Its mathematical formulation exposes the two-stage missing-data contract, dilated wavelet support, scale-normalized residual score, observation-support gate, quantile threshold, event metrics, resampling estimator, and complexity audit used by the implementation. The resulting detector is therefore defined by a reproducible score contract rather than by an architecture diagram alone.

Across six public sensor benchmarks, RC-WMRAD changes the balance among ranking, event coverage, and alarm burden rather than producing a uniform gain. SMD shows lower false alarms with lower event recall against Raw-TCN; MSL shows higher AUPRC and lower false alarms; and SMAP shows lower AUPRC and lower false alarms. On the physical MetroPT-3 railway asset, RC-WMRAD lowers alarm burden against Raw-TCN and TranAD-style, while TranAD-style ranks failures more strongly and responds earlier. USAD-style and GDN-style comparisons add reconstruction and graph-predictive boundaries without supporting a universal ranking claim. Robustness and calibration experiments identify effects that persist locally, but they do not justify extrapolation to untested faults or thresholds.

The contribution is consequently a reproducible conditional-effect study: an auditable multi-scale residual formulation, an evaluation chain that separates score ranking from the locked operating point, and explicit evidence boundaries. The industrial mapping specifies acquisition, causal context, calibration, alarm segmentation, and operator handoff while identifying the untested adaptation and edge interfaces. The current evidence supports further prospective testing, not a universal detector, a sole safety monitor, or a completed field-deployment claim. Entity-resolved evaluation, richer normal-only uncertainty estimates, broader verified comparators, and deployment measurements on the intended edge target remain necessary to convert these benchmark findings into a field-level systems claim. Complete per-dataset effect forests, entity-level detail, severity trajectories, calibration diagnostics, fusion-ablation counts, and the dataset-admissibility audit referenced throughout this manuscript are provided in the Supplementary Materials (Figures S1–S8).

## Figures and Tables

**Table 1:** **Dataset and protocol matrix.** A ✓ denotes a verified protocol property and a ✕ denotes absence from the admitted evidence. “Fixed threshold” means a predeclared quantile of independent normal-calibration scores; all reported evaluations use no point adjustment. SKAB units are profiled files pooled by its evaluator; SMD, PSM, MSL, and SMAP units are entities or channels; MetroPT-3 is one physical asset with four reported failure intervals.

Dataset	Units	Seeds	Normal Fit	Independent Cal.	Labels After Lock	Fixed Threshold	No PA	Robustness Grid
SKAB	35	5	✓	✓	✓	✓	✓	✕
SMD	28	5	✓	✓	✓	✓	✓	✓
PSM	1	5	✓	✓	✓	✓	✓	✓
MSL	27	5	✓	✓	✓	✓	✓	✕
SMAP	53	5	✓	✓	✓	✓	✓	✕
MetroPT-3	1	5	✓	✓	✓	✓	✓	✕

**Table 2:** **Model-component matrix.** A ✓ denotes a present and audited component; a ✕ denotes absence or, for fixed paths, the absence of an independently audited architecture. Symbols accompany the binary distinctions so interpretation does not depend on color.

Model or Score Path	Raw Path	Causal Wavelet Path	Mask History	Learned Fusion	Normal Threshold Lock	Parameters Audited
Raw-TCN	✓	✕	✓	✕	✓	✓
Fixed raw-only path	✓	✕	✓	✕	✓	✕
Fixed wavelet-only path	✕	✓	✓	✕	✓	✕
RC-WMRAD	✓	✓	✓	✓	✓	✓
TranAD-style	✓	✕	✓	✕	✓	✕

**Table 3:** **Formal cross-dataset effects.** The admitted descriptive SKAB seed range is retained. Entries are RC-WMRAD minus the named comparator. This descriptive SKAB observed seed range is different from Figure 3’s inferential paired-seed bootstrap interval; all other brackets are 95% bootstrap intervals using the unit in the final column. Pale teal marks a supported beneficial direction, pale coral a supported degradation, and neutral gray an interval containing zero. Signs and interval bounds provide redundant semantics. AUPRC was not admitted for the SKAB snapshot. No pooled estimate is computed.

Dataset	Comparator	ΔAUPRC	ΔEvent Recall	ΔFA/Hour	Statistical Unit and Interval
SKAB	Raw-TCN	–	0.0000 [0.0000,0.0000]	−0.1545 [−0.3259,−0.0430]	5 paired seeds; observed seed range
SMD	Raw-TCN	+0.0260 [−0.0257,+0.0810]	−0.2143 [−0.2729,−0.1534]	−0.1380 [−0.2812,−0.0357]	5×28 pairs; 95% hierarchical bootstrap
PSM	TranAD-style	−0.0372 [−0.1163,+0.0435]	−0.4056 [−0.6620,−0.1408]	0.0000 [0.0000,0.0000]	5 paired seeds; 95% seed bootstrap
MSL	Raw-TCN	+0.0380 [+0.0138,+0.0658]	+0.0667 [−0.0148,+0.1852]	−1.1103 [−1.7432,−0.5957]	5×27 pairs; 95% hierarchical bootstrap
SMAP	Raw-TCN	−0.0439 [−0.0817,−0.0094]	−0.0063 [−0.0403,+0.0201]	−0.2342 [−0.3330,−0.1560]	5×53 pairs; 95% hierarchical bootstrap
MetroPT-3	Raw-TCN	−0.0015 [−0.0143,+0.0154]	+0.0500 [−0.1500,+0.3000]	−0.5949 [−1.0684,−0.1034]	1 asset, 4 reports, 5 paired seeds
MetroPT-3	TranAD-style	−0.0109 [−0.0204,−0.0044]	−0.0500 [−0.1500,0.0000]	−0.5095 [−0.5944,−0.4403]	1 asset, 4 reports, 5 paired seeds

**Table 4:** **MetroPT-3 broader baseline comparison.** Entries are five-seed means at a fixed one-minute decision cadence. Every threshold is the model’s own normal-calibration $Q_{0.995}$; events remain the four company reports and no point adjustment is applied. Green cells mark the best descriptive mean only; inferential comparisons are reported in the text and remain paired by seed.

Model	AUPRC	AUROC	Event Recall	FA/Hour
RC-WMRAD	0.0412	0.7143	0.950	0.542
Raw-TCN	0.0427	0.6682	0.900	1.137
TranAD-style	0.0521	0.7954	1.000	1.052
USAD-style	0.0431	0.7490	0.800	0.542
GDN-style	0.0344	0.6755	0.400	0.563

**Table 5:** **Independently optimized SKAB component ablation.** Means use five independent seeds. Bracketed entries are 95% paired entity–seed bootstrap intervals for the variant-minus-A0 difference over 40 entities and five seeds. Green denotes a significant favorable difference, red a significant unfavorable difference, and gray an interval crossing zero. Thresholds are fitted independently from normal calibration scores; no point adjustment is used.

Variant	AUPRC	ΔAUPRC [95% CI]	Event Recall	FA/h	ΔFA/h [95% CI]
A0: Full RC-WMRAD	0.578	Reference	1.000	0.309	Reference
A1: Raw only	0.583	+0.009 [−0.026, +0.044]	1.000	0.490	+2.992 [+0.995, +5.532]
A2: Wavelet only	0.572	−0.004 [−0.011, +0.004]	1.000	0.261	−0.804 [−1.882, +0.124]
A3: Fixed 0.5 fusion	0.578	+0.004 [−0.003, +0.012]	1.000	0.314	+0.099 [−0.375, +0.592]
A4: Global gate	0.578	+0.000 [−0.000, +0.000]	1.000	0.309	+0.000 [+0.000, +0.000]
A5: No mask gate	0.578	+0.000 [−0.000, +0.000]	1.000	0.309	+0.000 [+0.000, +0.000]

**Table 6:** **Robustness summary across three severities per family.** Arrows show the direction of the mean RC-WMRAD-minus-Raw-TCN effect. The fraction before ✓ is the number of 95% intervals excluding zero; ✕ denotes zero supported intervals. Pale teal marks beneficial evidence, pale coral marks degradation, and neutral gray marks mixed or inconclusive evidence. Arrow, count, and symbol make the table interpretable without relying on color alone. SMD uses hierarchical seed–entity intervals; PSM uses paired-seed intervals for one entity.

Dataset	Corruption Family	Severities	ΔAUPRC	ΔEvent Recall	ΔFA/Hour
SMD	Noise	0.05/0.10/0.20	↑ 0/3 ✕	↓ 3/3 ✓	↓ 3/3 ✓
SMD	Missingness	0.05/0.10/0.20	↑ 0/3 ✕	↓ 3/3 ✓	↓ 3/3 ✓
SMD	Drift	0.05/0.10/0.20	↑ 0/3 ✕	↓ 3/3 ✓	↓ 3/3 ✓
SMD	Regime shift	0.10/0.25/0.50	↑ 0/3 ✕	↓ 3/3 ✓	↓ 3/3 ✓
PSM	Noise	0.05/0.10/0.20	mixed; 1/3 ✓	↑ 0/3 ✕	↑ 0/3 ✕
PSM	Missingness	0.05/0.10/0.20	↑ 3/3 ✓	↓ 3/3 ✓	↓ 2/3 ✓
PSM	Drift	0.05/0.10/0.20	↑ 1/3 ✓	mixed; 0/3 ✕	mixed; 0/3 ✕
PSM	Regime shift	0.10/0.25/0.50	↑ 1/3 ✓	mixed; 0/3 ✕	mixed; 0/3 ✕

**Table 7:** **Chronological normal-calibration size sensitivity on MetroPT-3.** Values are five-seed means. Each column pair reports event recall and false alarms per hour after fitting $Q_{0.995}$ from a nested prefix of the same normal calibration stream. Test scores remain frozen, labels are read only after threshold construction, and no point adjustment is used.

Calibration	RC-WMRAD		Raw-TCN		TranAD-Style	
Fraction (Rows)	Recall	FA/h	Recall	FA/h	Recall	FA/h
10% (532)	0.95	0.778	0.95	1.423	1.00	1.037
25% (1332)	0.95	0.743	0.95	1.166	1.00	1.023
50% (2664)	0.95	0.583	0.90	1.204	1.00	1.084
100% (5329)	0.95	0.542	0.90	1.137	1.00	1.052

**Table 8:** **Complexity audit.** Values are reported at width 32 and 38 variables and copied from the tracked model-complexity artifact. Forward FLOPs are a single batch-one profiler count and do not represent training cost. A ✓ denotes the common locked evaluation protocol.

Model	Trainable Parameters	Forward FLOPs, Batch 1	Normal Threshold Lock	No PA	Audit Boundary
RC-WMRAD	26,111	29,724	✓	✓	Parameters and forward FLOPs only
Raw-TCN	42,220	29,478	✓	✓	Forward-compute-matched control
TranAD-style	26,156	4,842,278	✓	✓	Parameter-comparable comparator

**Table 9:** **Measured batch-one inference efficiency.** Values are p50 [p95] milliseconds over 1000 calls after 200 warm-ups at L=128 and D=38. GPU calls are synchronized before each elapsed-time sample. End-to-end timing includes NumPy standardization, tensor construction and transfer, and model inference. CPU measurements use one thread. These local warm-start results do not measure energy, cold start, concurrent workloads, or sustained streaming I/O.

Model	CPU Model-Only	GPU Model-Only	CPU End-to-End	GPU End-to-End
RC-WMRAD	1.325 [1.670]	2.603 [3.155]	1.358 [1.723]	2.775 [3.560]
Raw-TCN	1.572 [1.973]	1.968 [2.460]	1.613 [2.072]	2.120 [2.559]
TranAD-style	1.402 [1.768]	1.309 [1.588]	1.456 [1.834]	1.432 [1.774]

**Table 10:** **Post-lock MetroPT-3 partial-event quality.** Coverage is the fraction of scored timestamps alarmed inside each company-reported interval and includes missed seed–report pairs as zero. Mean coverage uses all 20 pairs. Report 4 normalized delay is conditional on detection; the parenthetical entry for GDN-style records that only one of five seeds detected this report.

Model	Detected Pairs	Mean Coverage	Report 4 Median Coverage	Report 4 Normalized Delay
RC-WMRAD	19/20	0.357	0.330	0.631
Raw-TCN	18/20	0.448	0.359	0.631
TranAD-style	20/20	0.818	0.441	0.0067
USAD-style	16/20	0.258	0.378	0.0104
GDN-style	8/20	0.003	0.000	0.440 (1/5)

**Table 11:** **Industrial deployment-readiness boundary.** Verified entries are retrospective properties of the present data and audit. A ✕ marks field evidence that remains absent; it is not a statement that the corresponding engineering requirement is unnecessary.

Area	Current Contract or Measurement	Operational Interpretation	Field Status
Acquisition	15 channels at median 10 s; one physical asset	Timestamped values and observation masks	✓ Retrospective
Context and cadence	L=128 (about 21 min); one-minute decision cadence	Rolling causal history and one score per decision	✓ Retrospective
Calibration	Training-only median/MAD; normal-only $Q_{0.995}$; no point adjustment	Threshold provenance is fixed before labels	✓ Retrospective
Alarm interface	Contiguous positive segment as a review-task proxy	Requires persistence, routing, acknowledgment, and ownership	✕ Not field tested
Compute	Warm CPU end-to-end p50 1.358 ms; 30-s RC-WMRAD: 335.2 CPU and 179.5 CUDA requests/s; cold-start p50 3033.5 ms	Large local timing margin on the named workstation	✓ Workstation only
Adaptation	Model and threshold remain fixed during each test	Predefined prospective recalibration and drift triggers are unspecified	✕ Not field tested
Edge operation	Short workstation concurrency, ingestion, temperature, and power telemetry only; no embedded target	Hardware qualification, long-duration energy/thermal tests, and fail-safe integration remain required	✕ Not qualified

## Data Availability

The study uses the public SKAB v0.9, SMD, PSM, MSL, SMAP, and MetroPT-3 benchmarks cited in the manuscript. Derived evidence is represented by repository-local tracked audit artifacts and source-hashed figure manifests.

## Supplementary Materials

The following supporting information can be downloaded at: https://www.mdpi.com/article/10.3390/s26185751/s1, Figure S1: Complete per-dataset effect forests. RC-WMRAD-minus-comparator effects use paired-seed intervals for SKAB/PSM and hierarchical seed–entity intervals for SMD/MSL/SMAP. Units remain dataset-specific and are not pooled. Figure S2: Complete SMD entity effects. Five paired-seed effects are shown for each of 28 entities; horizontal ranges are descriptive minima and maxima, not confidence intervals. Heterogeneity precludes a common-effect interpretation. Figure S3: PSM severity trajectories. RC-WMRAD-minus-Raw-TCN effects use five paired seeds and 95% seed-bootstrap intervals with clean thresholds held fixed. One entity does not provide entity-level robustness evidence. Figure S4: SMD severity trajectories. RC-WMRAD-minus-Raw-TCN effects use five seeds, 28 entities, and 95% hierarchical intervals with clean thresholds held fixed. The tested grid does not imply robustness to unmeasured corruptions. Figure S5: Seed stability. Five paired RC-WMRAD-minus-Raw-TCN differences are retained separately for SMD and SKAB. Lines show seed-specific effects without intervals; pooled SKAB stability does not replace entity-resolved uncertainty. Figure S6: Calibration scores and locked thresholds. ECDFs summarize 67,269 normal-calibration scores per model and seed across five seeds. Markers are locked 99.5% higher-order quantiles; score distributions do not measure test performance. Figure S7: Fusion-ablation details. Exhaustive paired SMD event counts compare learned fusion with fixed raw-only and wavelet-only paths across five seeds and 28 entities. Post-primary rescoring is diagnostic, not a model ranking. Figure S8: Dataset admissibility and evidence audit. The panel records 13 acceptance checks, 33 verified submission files, 27 MSL channels, 53 admitted SMAP channels, and one excluded conflict channel. Audit completeness does not establish scientific superiority.
