# Supplementary material for: Causal Wavelet Residual Learning with Observation-Support Gating for Industrial Sensor Anomaly Detection
Source: Sensors (Basel). 2026 Sep 10;26(18):5751. doi: 10.3390/s26185751 (PMC13611024; doi:10.3390/s26185751)
Supplement: Supplementary file 1 [file sensors-26-05751-s001.zip › sensors-4504527-supplementary.pdf]

Supplementary Materials: Reliability-Calibrated Causal Wavelet Residual Learning for Industrial Sensor Anomaly Detection

Supplementary Figures

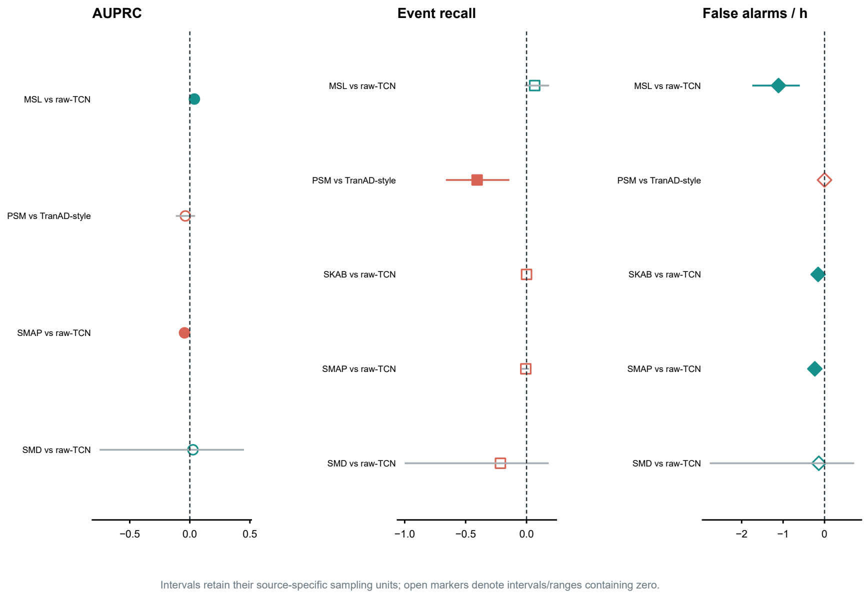

Figure S1. Complete per-dataset effect forests. RC-WMRAD-minus-comparator effects use paired-seed intervals for SKAB/PSM and hierarchical seed–entity intervals for SMD/MSL/SMAP. Units remain dataset-specific and are not pooled.

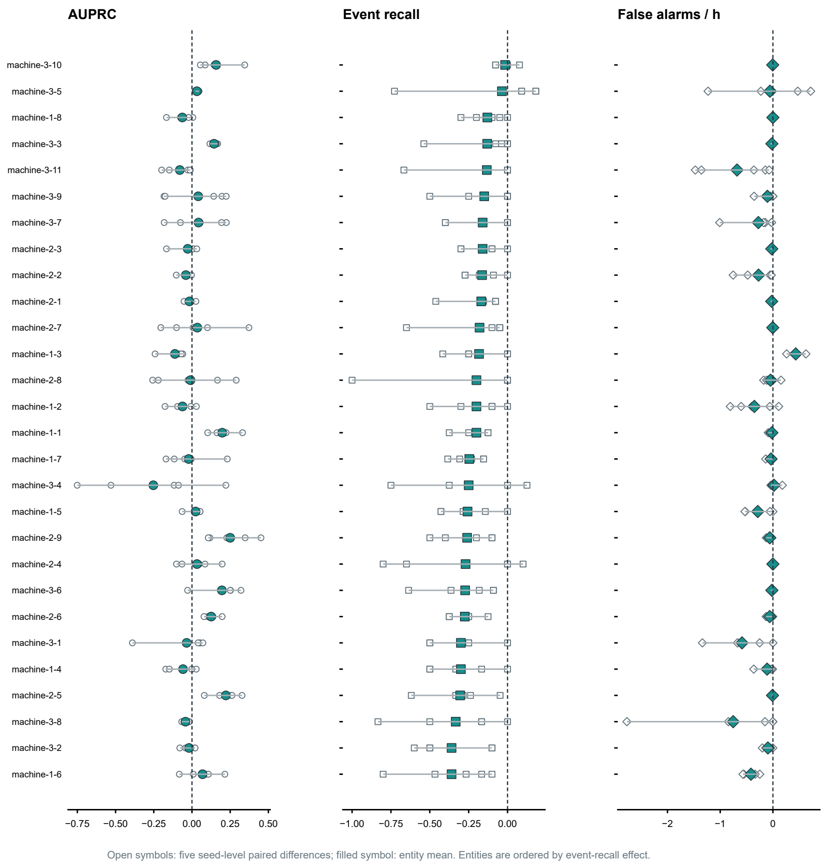

Figure S2. Complete SMD entity effects. Five paired seed effects are shown for each of 28 entities; horizontal ranges are descriptive minima and maxima, not confidence intervals. Heterogeneity precludes a common-effect interpretation.

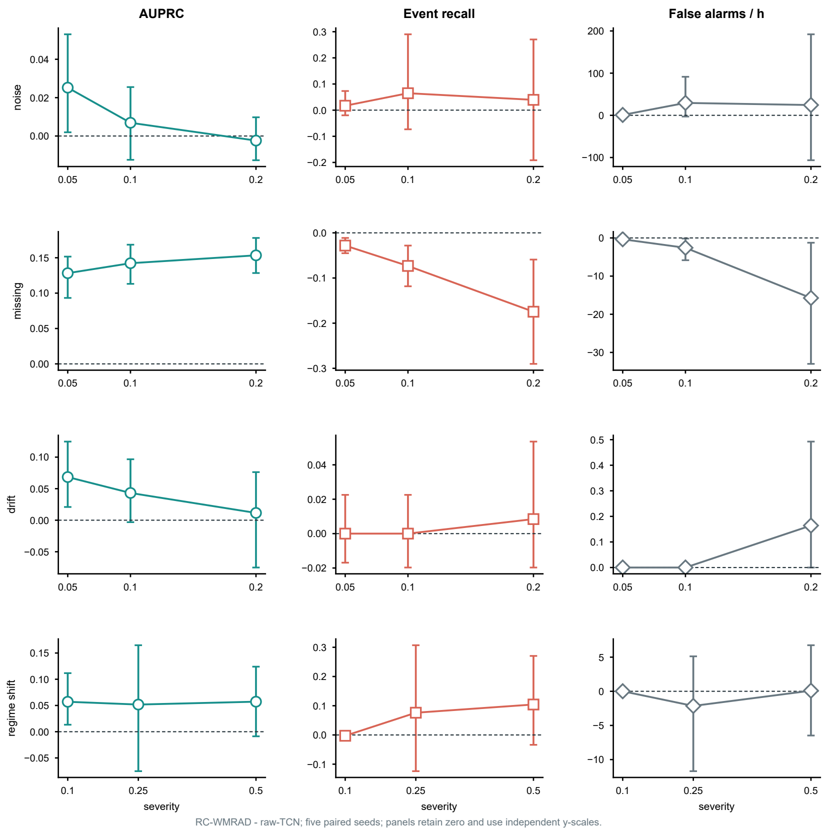

Figure S3. PSM severity trajectories. RC-WMRAD-minus-raw-TCN effects use five paired seeds and 95% seed-bootstrap intervals with clean thresholds held fixed. One entity does not provide entity-level robustness evidence.

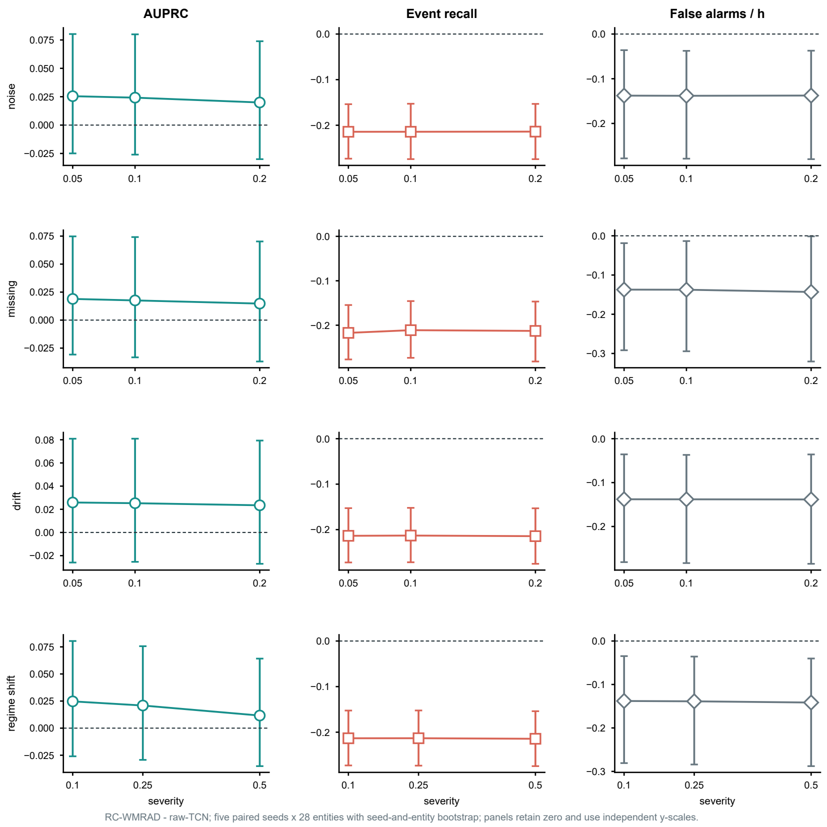

Figure S4. SMD severity trajectories. RC-WMRAD-minus-raw-TCN effects use five seeds, 28 entities, and 95% hierarchical intervals with clean thresholds held fixed. The tested grid does not imply robustness to unmeasured corruptions.

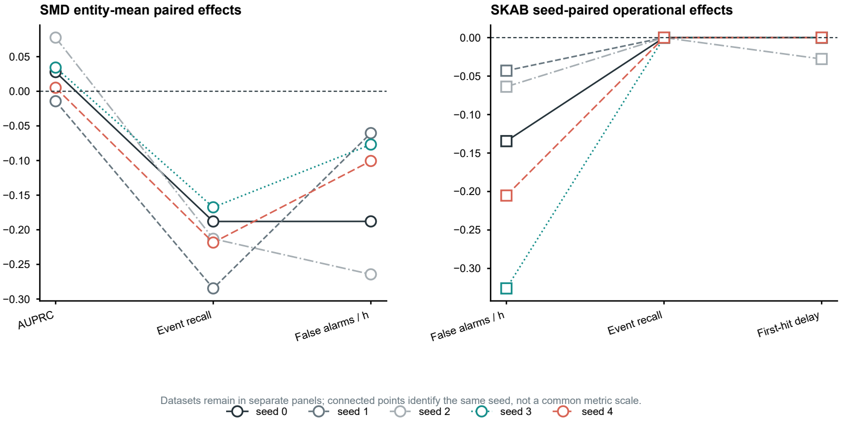

Figure S5. Seed stability. Five paired RC-WMRAD-minus-raw-TCN differences are retained separately for SMD and SKAB. Lines show seed-specific effects without intervals; pooled SKAB stability does not replace entity-resolved uncertainty.

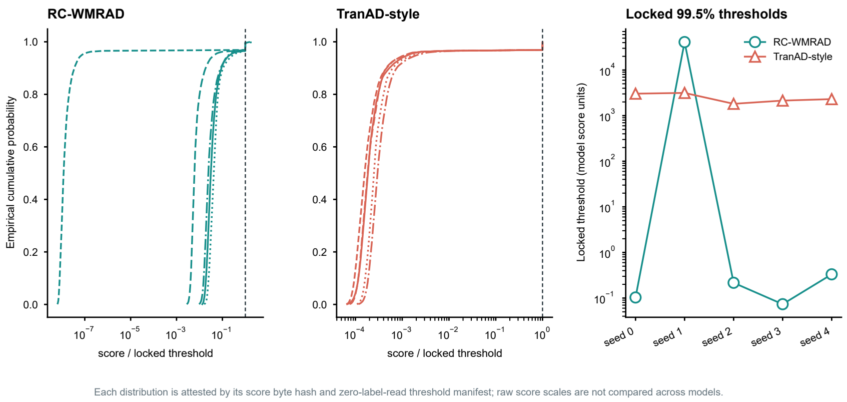

Figure S6. Calibration scores and locked thresholds. ECDFs summarize 67,269 normal-calibration scores per model and seed across five seeds. Markers are locked 99.5% higher-order quantiles; score distributions do not measure test performance.

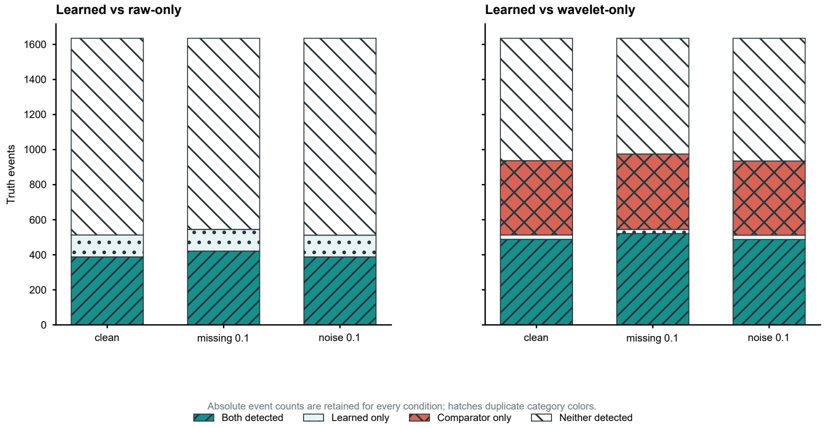

Figure S7. Fusion-ablation details. Exhaustive paired SMD event counts compare learned fusion with fixed raw-only and wavelet-only paths across five seeds and 28 entities. Post-primary rescoring is diagnostic, not a model ranking.

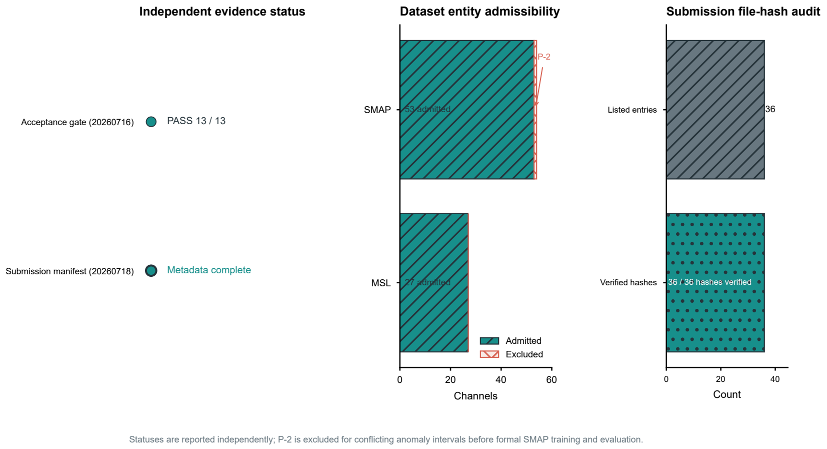

Figure S8. Dataset admissibility and evidence audit. The panel records 13 acceptance checks, 33 verified submission files, 27 MSL channels, 53 admitted SMAP channels, and one excluded conflict channel. Audit completeness does not establish scientific superiority.
